# Bovine Colostrum in Pediatric Nutrition and Health

**DOI:** 10.3390/nu16244305

**Published:** 2024-12-13

**Authors:** Ahmet Alperen Canbolat, Mauro Lombardo, Alicia del Carmen Mondragon, Jose Manuel Miranda López, Mikhael Bechelany, Sercan Karav

**Affiliations:** 1 Department of Molecular Biology and Genetics, Çanakkale Onsekiz Mart University, Çanakkale 17000, Türkiye; ahmetalperencanbolat@stu.comu.edu.tr; 2 Department for the Promotion of Human Science and Quality of Life, San Raffaele Open University, Via di 11 Val Cannuta 247, 00166 Rome, Italy; mauro.lombardo@uniroma5.it; 3 Laboratorio de Higiene Inspección y Control de Alimentos, Departamento de Química Analítica, Nutrición Bromatología, Universidade de Santiago de Compostela, Campus Terra, 27002 Lugo, Spain; alicia.mondragon@usc.es (A.d.C.M.); josemanuel.miranda@usc.es (J.M.M.L.); 4 Institut Européen des Membranes (IEM), UMR 5635, University Montpellier, ENSCM, CNRS, F-34095 Montpellier, France; mikhael.bechelany@umontpellier.fr; 5 Functional Materials Group, Gulf University for Science and Technology (GUST), Masjid Al Aqsa Street, Mubarak Al-Abdullah 32093, Kuwait

**Keywords:** colostrum, dairy foods, pediatric nutrition, disease, glycan, preterm infants, milk, necrotizing enterocolitis

## Abstract

Bovine colostrum (BC), the first milk secreted by mammals after birth, is a trending alternative source for supplementing infants and children, offering benefits for gut and immune health. Its rich components, such as proteins, immunoglobulins, lactoferrin, and glycans, are used to fortify diets and support development. Preterm development is crucial, especially in the maturation of essential systems, and from 2010 to 2020, approximately 15% of all premature births occurred at less than 32 weeks of gestation worldwide. This review explores the composition, benefits, and effects of BC on general infants and children, along with preterm infants who require special care, and highlights its role in growth and development. BC is also associated with specific pediatric diseases, including necrotizing enterocolitis (NEC), infectious diarrhea, inflammatory bowel disease (IBD), short-bowel syndrome (SBS), neonatal sepsis, gastrointestinal and respiratory infections, and some minor conditions. This review also discusses the clinical trials regarding these specific conditions which are occasionally encountered in preterm infants. The anti-inflammatory, antimicrobial, immunomodulatory, and antiviral properties of BC are discussed, emphasizing its mechanisms of action. Clinical trials, particularly in humans, provide evidence supporting the inclusion of BC in formulas and diets, although precise standards for age, feeding time, and amounts are needed to ensure safety and efficacy. However, potential adverse effects, such as allergic reactions to caseins and immunoglobulin E, must be considered. More comprehensive clinical trials are necessary to expand the evidence on BC in infant feeding, and glycans, important components of BC, should be further studied for their synergistic effects on pediatric diseases. Ultimately, BC shows promise for pediatric health and should be incorporated into nutritional supplements with caution.

## 1. Introduction

Bovine colostrum (BC) is the first milk secreted by the mammary glands of mammalian species after parturition and has a thicker texture than milk [1]. BC possesses diverse components, including macro and micronutrients, biological peptides, immunoglobulins, and growth factors, along with other ingredients that function primarily in antimicrobial activity [2]. The high nutritional value of BC provides a broad range of food and functional applications [3]. BC is crucial to neonates because of the presence of key nutrients essential for energy, development, and growth. If human milk is limited or unavailable, BC is often preferred as an alternative source of nutrients, such as glucose, amino acids, lactose, and proteins, for infants [4]. In addition to providing developmental support, BC offers constitutional immune protection to newborns, building their innate immunity until the adaptive immunity of neonates matures to match their specific environment.

The colostrum is essential for neonates and provides the nutrients required for essential vital activities. One of the vital functions of milk and colostrum for newborns is to prepare the innate immune system for external environmental conditions, resulting in the onset of adaptive immunity [5]. As an antimicrobial, immunological, and nutritional conditioner, colostrum transitions the newborn from the mostly sterile conditions of fetal life to diverse microbial exposures and nutrients from postnatal breast milk intake. In this context, humans and other mammals share common external environmental conditions and are exposed to similar microorganisms, such as microbes, fungi, and viruses, through the external surfaces of the body (such as the gut, skin, or lungs) [6]. The functional development of the gastrointestinal tract is driven by colostrum consumption in mammalian infants. A range of complex glycans are abundantly found in bovine milk and its products. Glycans are key components of milk glycoproteins and shape microbiota by selectively promoting the growth of beneficial bacterial strains, based on the results of previous research [7]. BC influences metabolism and the hormonal system in neonatal calves and infants [8,9,10]. The muscular and skeletal repair system is also supported by BC because of its rich composition of bioactive contents [11]. BC consumption prevents injuries and strengthens muscles, which are results of the positive impact of BC on muscle and bone development because of its growth factors [12]. In general, BC has been evaluated as a nutraceutical and has been used in clinical trials to analyze its antiviral and antibacterial potential, with the finding that the antimicrobial properties of colostrum from one species may be effective in another species [13,14,15]. Undoubtedly, colostrum is essential for the survival of newborns in distinct mammals, such as cows and goats, whereas for human infants, it is considered important but not vital for survival.

Both BC and human colostrum (HC) and their milk products have highly similar compositions, but there are considerable nutritional differences in terms of carbohydrate, lipid, mineral, protein, and vitamin concentrations along with bioactive components (i.e., immunoglobulins) [16]. Nonetheless, the precise and functional immunological support of dairy products in infants and children has not been fully elucidated. In BC applications, safety and tolerability for age groups, including neonates and infants as well as children with allergies, is the first emerging concern. Although prominent studies exist, the mechanisms of bioactive components are still not fully clarified. Current evidence and standard dosage and administration guidelines remain also insufficient in this regard. After optimizing milk products in terms of nutrition for infants and children, evidence from preterm infants in the following years has revealed several serious concerns about these milk products. According to affiliated studies, processed cow milk formulas cause more necrotizing enterocolitis (NEC), inflammatory bowel syndrome (IBD), sepsis (late-onset sepsis, LOS), food intolerance (FI), and allergies compared to either infant-fed formula or a combination with human milk [6,17,18,19,20,21]. The reason for the observable adverse effects of formula products in comparison with milk remains unclear, whether due to their bovine origin, the inclusion of vegetable products, or industrial processing (serial heat treatment or filtration). Additionally, it is not clear whether the risk conditions for infants vary between term and preterm infants, between bovine dairy products and those from different mammalian species (i.e., camels, donkeys, and goats), or between colostrum and milk [6]. New approaches for the production of hypoallergenic infant formulas are important factors in infant nutrition and are currently being investigated. Commercially available formulas frequently promote classical symptoms of cow milk allergy (CMA), and one of the most commonly applied methods to overcome related allergic reactions is the elimination of these allergens from the infant diet; however, the most crucial allergens of milk products are milk proteins, and alternative compensation methods must be investigated since the isolation of milk proteins severely decreases the benefits of milk products [22]. BC is also present in these milk products and should be investigated in this regard.

This review covers the significance, applications, and participation of bovine colostrum (BC) in pediatric conditions and diseases, focusing on its nutritional, preventive, or therapeutic properties for supplementation in severe pediatric or immunologic diseases, particularly in premature infants. The review also comprises clinical animal models and specific human studies. The extent of colostrum in the context of utilization, risk factors, and specific diseases are discussed in detail. Additionally, glycan, one of the most important and common components of colostrum and dairy products, is discussed in detail, as have its applications under several conditions.

## 2. Components of Colostrum

**Figure 1:**
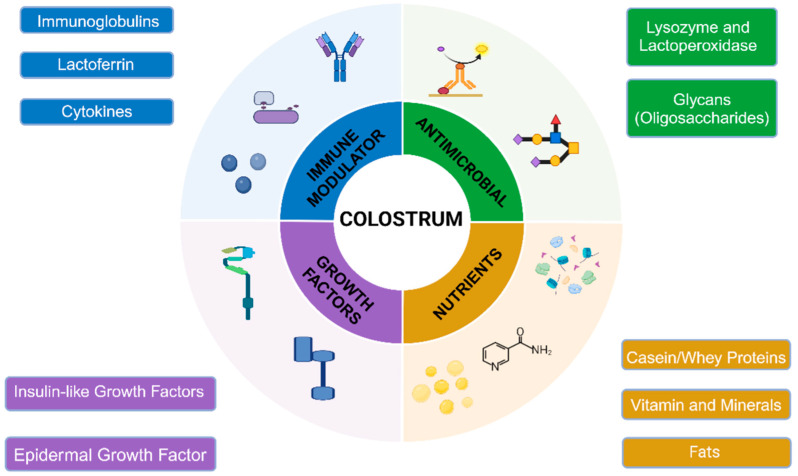
The key components of bovine colostrum. The antimicrobial and immune-modulating properties, growth factors, and nutrients of BC are essential for infants. The relative concentrations of BC differ daily and transform into milk 7 days after parturition (created with BioRender.com, accessed on 27 August 2024).

The composition of BC is close to that of mature bovine milk products; the only difference is the variable rates of bioactive components, making BC a key component for biological processes (Figure 1). BC offers a rich compositional similarity which makes BC a suitable alternative source compared to HC [23]. BC includes higher amounts of IgG content, growth factors, and protein content, promoting muscle growth, tissue repair, and adaptive immunity (see later sections). Although BC is less species-specific than HC, its more abundant contents result in the particularly effective general wellness and recovery of essential systems. Also, the abundance of BC over HC should be noted in the context of availability. Since essential developmental progress in preterm infants is often insufficient, the benefits of BC may aid in their improvements and adjust the insufficient systems when properly used.

### 2.1. Macronutrients

**Figure 2:**
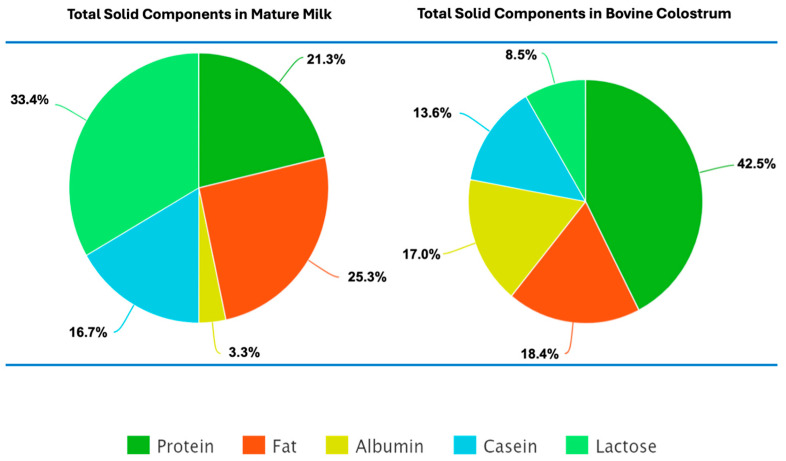
This chart shows the mean (±) levels of total solid components present in BC and mature milk [2]. The difference between BC and mature milk indicates that while BC supports immunity and growth in newborn infants, mature milk is prevalent in terms of its nutritional ingredients. (Created with meta-chart.com, accessed on 24 August 2024).

Both human and bovine colostrum consist of analogous structures and components in terms of their nutritional ingredients; the only difference between them is the relative concentration of major macronutrients, in which the protein and fat contents of BC are highly concentrated [23]. At the onset of lactation, the protein and fat concentrations of colostrum are relatively higher than those of lactose, but over time, the protein and fat concentrations decrease as lactose increases [24]. As the lactose content of milk increases over time and as offspring mature, the immunological and trophic role of colostrum transforms into a more nutritional role, ultimately becoming milk [25]. As time passes, the contents of colostrum decrease in favor of nutritional properties other than protective properties. The total carbohydrate composition of colostrum and milk also includes oligosaccharides, which have larger molecular structures than mono- and disaccharides do [6]. As mentioned above, the protein concentrations of BC and milk are much greater than those of HC and milk. This difference also leads to key differences in amino acid concentrations and availability (Figure 2) [6,25].

Milk proteins are generally classified as whey or casein proteins, and throughout mammalian species, their total protein fractions vary [26]. In bovine milk, casein proteins make up 80% of the total protein, and whey proteins make up 20% [27]. In contrast, overall, human milk is dominated by whey proteins (60%) rather than caseins (40%) [28]. Further inspection of milk protein fractions revealed that the milk composition of this species is highly homologous. Specifically, the most common and studied bioactive components of colostrum are immunoglobulins (Igs), lactoferrin (LF), lysozyme, α-lactalbumin, and growth factors [6]. Colostrum contains growth factors such as epidermal growth factor (EGF), transforming growth factor β (TGF-β), platelet-derived growth factor (PDGF), and insulin-like growth factor (IGF), which contribute to wound healing and cell proliferation [29]. The nutritional value of colostrum stems from both its amino acid content and how its proteins are digested in the stomach and upper intestine [30]. Casein proteins create a gel-like structure (or clots) in the upper intestinal compartments, slowing the release of amino acids for absorption, whereas whey proteins dissolve more rapidly and are transferred to the small intestine for faster digestion and absorption [6]. Whey proteins are resistant to protease enzymes, which degrade proteins, and this ability keeps whey proteins functional throughout the GI tract. The variations in resistance levels depend on the age of the infant, as preterm infants are not able to produce enough gastric acid and digestive enzymes. As a result of this lack, preterm infants on breast milk absorb fewer beneficial proteins in the GI tract. It can be assumed that colostrum proteins are not intended merely for growth. The unique feature of colostrum also provides essential immune protection [31]. The diverse components of colostrum affect almost every compartment of the system. According to the current literature, several studies have investigated the potential benefits of whole BC and its distinct components in susceptible newborn infants, as well as their therapeutic effects on pediatric diseases.

### 2.2. Lactoferrin

Lactoferrin is a glycoprotein that is specifically capable of iron binding and is present in various biological fluids. The highest concentration of LF is found in milk, which means that HC is a valuable source of LF at even higher concentrations. Compared with milk, which has a concentration of 1 mg/mL, HC has an LF concentration ranging from 5 to 6 mg/mL [32]. BC, on the other hand, also includes LF at concentrations ranging from 1.5 to 5 mg/mL, which decreases from 0.02 to 0.35 mg/mL in bovine milk [33]. Some of the remarkable capabilities of LF are its antimicrobial, antioxidant, antitumor, and antiviral properties [34,35]. According to previous studies, LF performs its cellular functions via LF receptor (LFR) interactions. These receptors are located mainly in the microvilli of the intestinal cell membrane, maintaining their presence significantly in the jejunum during the early months of life, as observed in piglet models for the first time [36]. As soon as LF binds to the LFR, it is able to translocate into the cell nucleus, where it triggers gene transcription, resulting in increased cellular proliferation within the intestine [37]. Additionally, LF is capable of binding and sequestering iron (a crucial nutrient for both commensal and pathogenic bacteria), thereby enhancing its antimicrobial properties [6]. This selective antimicrobial ability allows for the elimination of pathogens and stimulates the growth of beneficial microorganisms such as *Lactobacillus* and *Bifidobacterium* in the GI tract [38]. Additionally, in another study, the selective deglycosylation pathway of LF affected the extent of the antimicrobial activity of LF. Sialylated glycans are responsible for the antimicrobial activity of LF, whereas neutral glycans do not have any significant effect [39]. It was also shown that bovine LF (BLF) stimulated the growth of *B. infantis* and *B. brevis*, whereas human LF (HLF) stimulated the growth of *B. infantis* much more effectively [40]. Both the promoting and hindering mechanisms of LF contribute to beneficial health effects, especially in the GI tract, and affect the whole organism.

Similarly, BHL and HLF share approximately 69% of their amino acid identity [41]. Owing to this high homology and the costs of BLF being much lower than those of HLF, BLF has been the most investigated LF species in clinical in vivo trials. Several studies have explored the potential of BLF in preventing inflammatory diseases in premature infants, including necrotizing enterocolitis (NEC) and late-onset sepsis (LOS) [42]. Randomized controlled trials have shown that daily supplementation with BLF in 472 infants or recombinant human LF in 120 infants can protect against NEC and LOS. However, the trial with the largest scale to date included 2203 infants, and LF failed to exert any protective effect [42,43]. A Cochrane meta-analysis with 12 randomized clinical trials and 5425 participants revealed low-certainty evidence that LF supplementation in enteral feeds decreases the LOS but not the NEC in preterm infants [44]. LF also has consistent treatment effects on viruses such as SARS-CoV-1. In addition, it has a special role in recovery because of its benefits to the overall immune system [45]. The vulnerability of preterm infants to respiratory system diseases is also a systemic and severe condition requiring special care, and LF has elimination and recovery effects, indicating its importance in viral infections. LF may support protective mechanisms and trigger the onset of immune response repair. LF also plays a role in dopaminergic cell mechanisms by hindering the Fenton reaction, causing damage with the help of its relative oxygen species (ROS)-modulating properties [46].

### 2.3. Lysozyme and α-Lactalbumin

**Figure 3:**
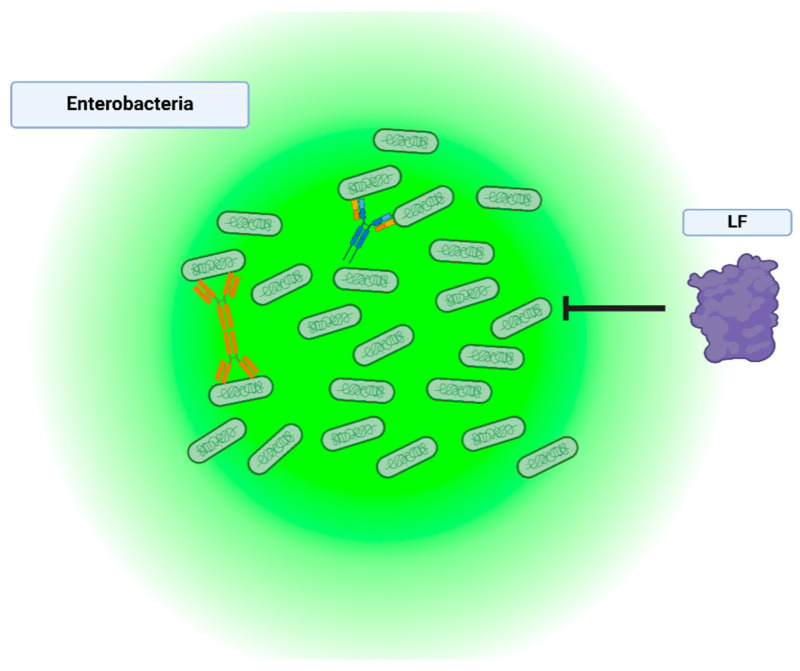
The hindering mechanism between *Enterobacteriaceae* and specific colostrum components. The figure illustrates the regulation of *Enterobacteriaceae* species in the infant gut, including pathogenic bacteria such as *E. coli* and *Salmonella*. Its bioactive components, particularly immunoglobulin A (IgA) and lactoferrin (LF), have strong antimicrobial properties. IgA binds to pathogenic bacteria, neutralizing them and preventing their attachment to the gut lining, whereas LF inhibits bacterial growth by sequestering iron, a nutrient essential for these bacteria. (Created with BioRender.com, access date: 7 September 2024).

Lysozyme is an enzyme belonging to the glycoside hydrolase family and is abundant in animal species. Owing to its antimicrobial properties (Figure 3), it plays a role in innate immune system functions and is found in various body secretions, including tears, saliva, human milk, and mucus [47,48]. This enzyme is broadly distributed in body fluids, as mentioned above, but is found at high concentrations in human breast milk, ranging from 200 to 400 μg/mL, whereas it is present at much lower concentrations in bovine milk, ranging from 0.05 to 1.5 μg/mL [6]. Some studies have reported that lysozyme promotes the protection of the intestinal wall by inhibiting the growth of harmful microorganisms (primarily bacteria) in the GI tract [49]. It also contributes to maintaining a healthy balance in the gut microbiota, which is essential for proper digestive functions and overall gut behaviors.

*α*-Lactalbumin is a whey milk protein that plays an important role in lactose synthesis in the mammary glands and serves as a source of bioactive components and amino acids that aid in infant development. Many biochemical and nutritional studies have revealed that α-lactalbumin is an important factor in early infant development [50]. Its concentrations vary from 1.2 to 1.5 mg/mL in bovine milk. Owing to its unique amino acid composition, especially tryptophan, lysine, sulfur-containing amino acids, and branched-chain amino acids, this protein is also believed to contribute to the infant intestinal system and brain development [51]. An improvement in the amino acid composition of bovine milk and its products can be achieved by adjusting the levels of α-lactalbumin and β-lactoglobulin in bovine milk to more closely match those in human milk [6]. Studies investigating the effects of α-lactalbumin also show that this protein has antibacterial potential [52]. In a study attributed to this property, α-lactalbumin, isolated from camels in its apo form, exhibited antibacterial effects on a bacterial strain named *P. aeruginosa* [53]. The isolated form of this protein may not be efficient against most harmful microorganisms, but a combination with enhancers may increase its effectiveness, and further studies should be designed to reveal its potential effects [52,54].

### 2.4. Immunoglobulins

The immunoglobulin distribution in BC consists of three distinct immunoglobulins, namely IgG (also divided into two subisotypes, IgG1 and IgG2), IgA, and IgM, and bovine milk also contains minor amounts of these immunoglobulins [31] (Table 1). Compositionally, approximately 90% of BC immunoglobulins are IgG, whereas the remaining immunoglobulins are IgM, IgA, and IgG2 [13,55,56]. The primary function of immunoglobulins is to provide essential immune system compartments for the survival of calves by hindering harmful microorganisms, including bacteria, microbes, and viruses [31]. These immunoglobulins are crucial for the survival of calves since without intake, calves are susceptible to pathogenic infections, leading to a high level of morbidity and mortality. Interactions among BC components are not unique to calves but may also have effects on various mammalian species [6]. Immunoglobulin studies indicate that the function and transport of immunoglobulins are not species-specific when BC effects are considered [57,58]. Calves and other mammalian species are born without immunoglobulins, and proper feeding of colostrum is thought to be necessary for the establishment of an immunoglobulin supply; the absorption of immunoglobulins is nonspecific in the first 12–36 h after parturition [59,60]. For preterm infants, the importance of immunoglobulins is much greater since they are developed insufficiently and need special care. Immunoglobulins are a major source of immune system functions. Colostrum feeding and absorption are more important for activating immunity and eliminating disease agents in the first weeks after birth [61]. The administration of colostrum just after birth helps calves eliminate infections such as *E. coli* [62].

### 2.5. Milk Fat Globule Membranes

Lipid droplets containing triacylglycerols are secreted by integration with the plasma membrane of alveolar epithelial cells, where they acquire a bilayer membrane composed of lipids and proteins, which is referred to as the milk fat globule membrane (MFGM) [6,63]. The bioactive ingredients of these droplets have antimicrobial, anti-inflammatory, and anticarcinogenic properties [64,65]. MFGM proteins are suggested to have nutritional and systemic benefits by assisting in the formation of healthy microbiota and exhibiting anti-infectious and anti-inflammatory properties [66,67]. Environmental factors, maternal genetics, gestation and lactation periods, body composition, and diet have radical influences on the composition of the MFGM [63]. The total MFGM content of BC, which is an abundant source of bioactive proteins, is attributed to the health of neonatal calves, indicating that it may be beneficial for preterm infants [68]. Bovine and human MFGMs are structurally and functionally similar; both have polar lipids in comparable amounts in both species. The lipid groups present in the MFGM are mainly phospholipids and sphingolipids. The MFGM proteome is also highly homologous, with some proteins being more abundant in the bovine MFGM [6].

MFGM proteins show very diverse and beneficial effects on human systems. Research has shown that adding MFGM-enriched protein fractions to milk and formula can aid in protection against infections [69]. For the prevention of diarrhea, certain MFGM proteins in whey protein concentrate inhibit pathogens caused by bacterial or viral strains [70]. In the context of neurobiology, studies involving premature infants indicate that sphingomyelin-fortified milk induces neurobehavioral development [71]. Additionally, sphingolipids such as gangliosides play crucial roles in neurodevelopmental pathways [72,73]. The MFGM proteome has diverse bioactivities, ranging from the antimicrobial functions of mucins and xanthine oxidase to the anticancer properties of fatty acid-binding proteins (FABPs) [74,75]. Mucins and xanthine oxidase act as decoys in the GI tract, preventing pathogens from interacting with epithelial cells. In addition, xanthine oxidase generates ROS and reactive nitrogen species (RNS) that have bactericidal effects. In the context of antiviral activity, one of the components of bovine whey protein with a high molecular weight exhibits antiviral effects on rotavirus in vitro [68]. MFGM glycoproteins are also considered potentially effective against other viruses, such as HIV [76]. When the composition of MFGM proteins is considered, butyrophilin is the most abundant protein and plays several roles in immune-modulation activities, namely, anti-infectious and anti-inflammatory activities [77,78]. Another point to consider is that MFGM supplementation in formula-fed infants has been shown to reduce the presence of *Moraxella catarrhalis*, a common pathogen causing ear infections in young children [79]. Considering the biodiversity of MFGM proteins and their affiliated properties, infant formulas supplemented with these proteins have great potential, leading to more complex and complementary feeding. For preterm infants, more precise and certain studies and trials are essential, yet the potential effects of MGFM proteins provide a trustworthy potential pool for the supplementation of formulas.

### 2.6. Glycans (Oligosaccharides)

**Figure 4:**
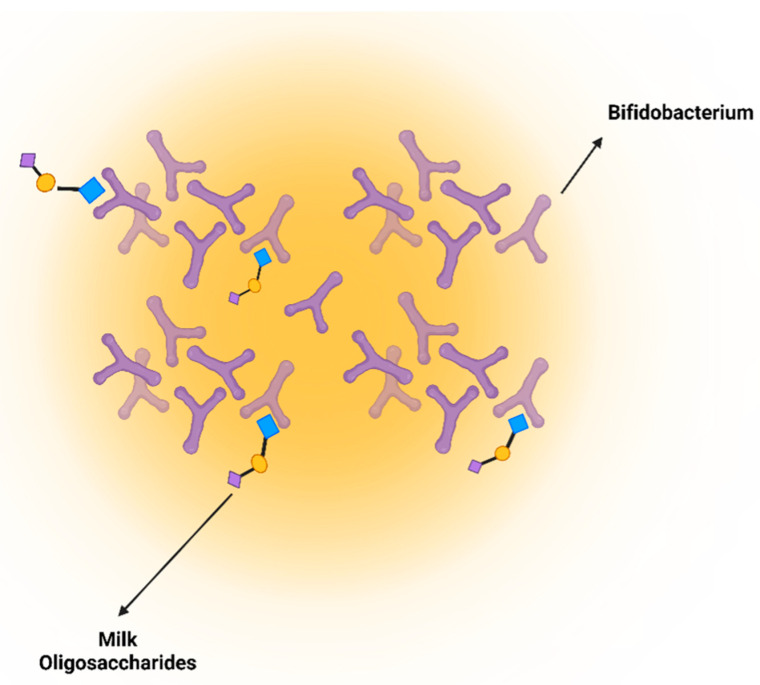
The interaction between milk oligosaccharides and Bifidobacteria species. Human milk oligosaccharides (HMOs), such as those found in human or bovine milk and colostrum, play a critical role in promoting the growth of *Bifidobacterium* in the infant gut, acting as selective prebiotics. *Bifidobacterium* ferments these oligosaccharides, producing short-chain fatty acids (SCFAs) such as acetate and butyrate, which help maintain gut health by lowering the pH and inhibiting pathogenic bacteria. This interaction also enhances the gut barrier, preventing harmful microbes from entering the bloodstream and supporting immune system development.

The carbohydrates present in colostrum are predominantly lactose, and several oligosaccharides have more than three monosaccharides structurally [80]. The core structure of oligosaccharides is either lactose or *N*-acetyl lactosamine [81]. Their classification is determined by the presence of a sialic acid, and they can be neutral or acidic [82,83]. A variety of oligosaccharides are present in bovine milk and colostrum, and different types of monosaccharides are bound to the core lactose of *N*-acetyl lactosamine [84]. Carbohydrates exist as free saccharides (such as glucose [Glc], milk oligosaccharides, and glycosaminoglycans [GAGs]) or as glycoconjugates (such as glycoproteins, proteoglycans, and glycolipids) [85,86]. Proteoglycans contain conjugated GAGs in their structure. Heparin, heparan sulfate (HS), and chondroitin sulfate are crucial for the immune system. The most important difference between proteoglycans and glycoproteins is the nature of the glycan [87]. Additionally, oligosaccharides are not digested in the upper GI tract; instead, they are fermented by gut microorganisms in the small intestine and colon [83,88]. The selective prebiotic activity of glycans makes them unique in shaping the composition of the gut microbiome (Figure 4) [89]. Colostrum has the highest levels of oligosaccharides, and after delivery, the levels diminish gradually [88]. Oligosaccharides are mostly abundant in human milk, ranging from 7 to 10 g/L, accounting for 10% of the total calories of milk. In HC, the amount of oligosaccharides increases to 22–24 g/L (1 g/L in BC) but decreases gradually at 48 h postpartum [80]. In a dedicated study by Karav et al. (2016), BC whey proteins were also reported to cleave a significant amount of complex milk glycans that are active biologically, indicating that the variability of glycans is very diverse and unpredictable [7].

GAGs consist of repeating disaccharide units with hexosamine and with diverse acetylation and sulfation activities, along with either uronic acid or galactose (Gal). However, glycoproteins are proteins with glycans that sequentially consist of one residue at a time, not repeating units. The glycosylation of proteins involves several kinds of modifications, including asparagine (*N*-linked glycans), *O*-GalNAc (*O*-linked *N*-acetylgalactosamine), or mucin-type glycans, and, finally, smaller glycan modifications, such as *O*-GlcNAc and *O*-fucose. *N*-glycans play roles particularly in protein folding quality control in the ER, whereas *O*-GlcNAc modifications play roles in signaling modulation, which is complementary, competes with, and acts independently from phosphorylation itself. Most importantly, *O*-fucose, or fucose (Fuc), which is located on serine or threonine residues, is crucial for lymphocyte development through the Notch pathway [87]. These fucosylated oligosaccharides provide developmental support for the nervous system and its healthy functions [90].

The microbiota can also synthesize glycans and glycoconjugates (e.g., peptidoglycan, lipopolysaccharides, and glycoproteins) [85,86]. As a key example, *Campylobacter jejuni* is capable of functioning via a basic *N*-linked glycosylation pathway, even though its glycan structure does not share a mammalian structure and composition. Another microorganism, *Mycobacterium tuberculosis*, can synthesize various glycan molecules, such as lipoarabinomannan, in its outer wall, neutralizing oxidizing agents. Fungi, on the other hand, synthesize *β*-glucans that target pattern recognition receptors (Dectin-1 and Dectin-2, which are part of the *C*-type lectin family (CTLF)). Additionally, viruses are also related to the production of glycans and are essential for mammals because of their viral life cycle. Viral proteins and their conjugated molecules are synthesized by the host cell replication machinery; thus, viral glycan structures generally mimic the glycome of infected cells [87].

Glycans are essential components of anti-inflammatory pathways in which tumor cells evade immune surveillance to protect themselves. The complexities of these mechanisms have been discovered through glycomic studies of autoimmunity disorders such as rheumatoid arthritis (RA). In a case study conducted in 1985, almost 1400 IgG-derived oligosaccharides from RA patients indicated that IgG glycans changed with the progression of autoimmune disease [91]. RA is related to a relative loss of galactose (Gal) and the exposure of terminal GlcNAc branching points in IgG *N*-glycans. Interestingly, female RA patients who were pregnant achieved remission of RA and had normal IgG glycosylation and sialylation during pregnancy [92]. However, after giving birth, these female subjects lost IgG Gal and sialic acid once again [93]. In addition to RA, certain autoimmune diseases also show significant changes in antibody glycosylation pathways. For example, increased IgG4 fucosylation has been detected in Hashimoto’s thyroiditis patients [94,95]. Crohn’s disease displays a correlation between the loss of IgG galactosylation and the severity of the disease [96]. In IgA nephropathy, on the other hand, the decrease in *O*-GalNAc glycan galactosylation within the hinge region of IgA1 heavy chains is closely associated [97]. In a different context, Sjögren syndrome triggers the loss of IgG sialylation, as in RA. Studies have shown that patients with allergies exhibit increased levels of sialic acid in total IgE [98]. Research by Shade et al. (2020) revealed that desialylation, the removal of sialic acid, reduced both effector cell degranulation and anaphylaxis in model organisms. Notably, the specific effect of sialic acid on IgE is poorly understood [99]. The colostrum, which is rich in bioactive molecules such as antibodies, immunoglobulins, and immune factors that can help support the development of the immune system in children, has potential therapeutic effects on related autoimmune diseases. These mechanisms include the modulation of immune responses, the improvement of antibody glycosylation, the promotion of gut health, and a reduction in inflammation. This situation may indicate that colostrum may be beneficial for managing the immune dysregulation associated with these diseases. The immunomodulatory and anti-inflammatory properties of colostrum may restore normal glycosylation patterns and mitigate disease symptoms; however, further investigation is needed to reveal the clinical benefits and optimal use of colostrum.

It remains uncertain in many situations whether changes in antibody glycosylation are merely indicators of disease, play a role in disease causation, or influence disease modulation. In the context of RA, the loss of sialic acid and Gal on IgG is not only indicative of disease but also that the proinflammatory nature of a galactosylated IgG actively contributes to the progression of the disease [100]. Notably, high-dose intravenous immunoglobulin (IVIg) therapy, which delivers sialylated IgG, effectively suppresses autoimmunity, demonstrating that the mechanism of action for IVIg therapy involves, at least partially, changes in IgG glycosylation [101,102].

The altered glycan theory is prompted by profound modifications in glycosylation pathways associated with inflammation, infection, and autoimmunity [103]. According to this theory, any form of autoimmunity creates a specific and unique glycan fingerprint on the basis of the relative presence of various glycoforms on tissues, cells, and glycoproteins. In summary, the altered glycan theory provides a framework for the exploration of glycosylation changes in autoimmune diseases, including those affecting children [104]. By applying this theory, researchers may identify unique glycan biomarkers for the early detection, monitoring, and treatment of pediatric autoimmune conditions such as T1D, JIA, pSLE, and autoimmune hepatitis. These advances might hold promise for improving pediatric autoimmunity management through targeted therapeutic and diagnostic innovations.

As mentioned, colostrum can colonize beneficial bacterial species in the colon. In a study by Karav and Mills, *B. infantis* exhibited significant growth by consuming released *N*-glycans as the sole carbon source from milk, although *B. lactis* did not exhibit any growth due to the lack of homologous enzymes. Infant-associated *Bifidobacterium* species, such as *B. infantis*, *B. breve*, and *B. bifidum*, are able to consume these released *N*-glycans and vastly colonize the colon [105,106]. In addition to their symbiotic effects, HC and BC may also be able to function as competitive inhibitors for pathogenic and harmful bacteria by hindering their binding to mucosal surfaces of the GI tract, resulting in the protection of neonates from bacterial infections [6,83]. Human milk oligosaccharides (HMOs) have also been proven to have anti-infectious effects against a wide range of pathogenic microorganisms, such as *Helicobacter pylori*, *Neisseria meningitidis*, and influenza virus, in a variety of models [107]. Recent studies have investigated the potential roles of HMOs in the protection of preterm infants from necrotizing enterocolitis (NEC). In a study using rodent models, the results indicated that the protective effects of HMOs are highly specific to their molecular structure [108]. The structural and prosperous specificity of oligosaccharides highlights the necessity for versatile and further research on milk oligosaccharide supplementation in infant formulas, specifically for vulnerable preterm populations.

## 3. Bovine Colostrum in the Context of Growth, Development, and Immune Function

**Figure 5:**
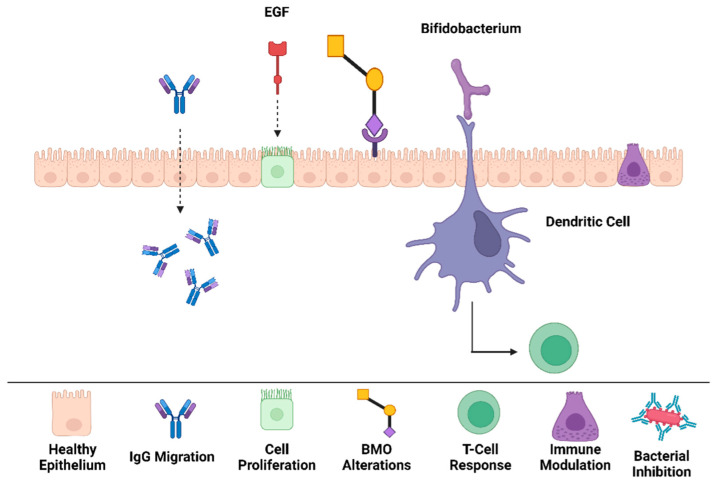
The biological effects of BC and its mechanisms of action. BC supports a healthy epithelium and administers immunoglobulins and growth factors to support immunity and induce cell proliferation. Milk oligosaccharides strengthen the gut barrier by promoting the development of the gut lining. The colostrum also contains immunoglobulins that help prime dendritic cells (DCs). Antibodies can bind to pathogens, making them easier for DCs to recognize, process, and present as antigens to T cells. This mechanism enhances the ability of the immune system to establish an effective response to infections in newborn infants. (Created with BioRender.com, accessed on 7 September 2024).

The nutritional requirements of infants (6 months and above) are fulfilled by designed and developed follow-up or growing-up formulas. The necessity of the growth, development, and establishment of a long-term diet can be provided by the complementary feeding period, especially when solid foods are introduced into enteral pathways alongside a milk-based diet. Milk from various species (mostly bovine, buffalo, camel, and goat) is utilized to produce dairy products such as fermented milk, kefir, cheese, and yogurt. These products are rich in bioactive components and nutritional benefits and are utilized in infant diets for different durations [109]. Infants have high vitamin and mineral requirements, particularly for iron and zinc. In recent decades, numerous modifications have been made to conventional infant formulas to better match the nutrient content of human milk [6]. These changes reduce overall protein and casein contents, increase α-lactalbumin, and lead to the use of essential oils instead of bovine milk lipids. The purpose is to mimic human milk, which is uniquely tailored to the requirements of infants and, in fact, is even individualized between a mother and her infant. To achieve this goal, novel ingredients such as lactoferrin, osteopontin, lutein, oligosaccharides, MFGMs, and essential fatty acids have been added to manufacture humanized formulas [110]. Despite these efforts, the replication of the exact nutritional and bioactive properties of human milk has proven to be harsh, primarily because of the instability and heat sensitivity of the bioactive components of colostrum. However, since breast milk or alternative milk-based formulas provides most of infants’ vital requirements, complementary foods should be nutrient-dense and diverse, including animal-source foods [6]. At this point, colostrum may be a powerful formula component, because as colostrum ingredients meet the essential requirements for infants, there may be a growing trend in the exploitation of colostrum in novel formulations (Figure 5).

Current studies suggest that BC should be used as a supplement for infants under certain conditions, such as the optimal age, time, and standardization for efficient and safe use [111,112,113]. BC can protect against gastrointestinal diseases, i.e., rotaviral diarrhea, necrotizing enterocolitis, sepsis, and chemotherapy-induced mucositis (all of these diseases will be discussed in detail in the next sections), specifically after pasteurization, and can be used as a supplement to infant formulas [114,115]. Considering the significant promise of BC, infant formulas can be fortified with BC or its components. The promotion of the gastrointestinal tract, the enhancement of the absorption of nutrients, and the strengthening of the defense mechanism of infants may be possible with the fortification of formulas with BC. It is also crucial to standardize the composition of BC fortifiers and design sufficient clinical trials to establish their safety and effectiveness before their introduction into preterm infant nutrition diets. Even if BC is beneficial because of its immunoglobulins and bioactive factors along with other components, it is not recommended for use as a sole nutritional source for infants because of nutritional imbalances compared with human or bovine milk. Adverse effects are minimal in the case of appropriate nutritional guidelines for infants; in fact, these guidelines may support therapies such as probiotics for immunocompromised children [6]. However, BC supplementation is recommended only when a mother’s own milk or donor milk is unavailable or insufficient to feed infants.

Bovine colostrum is extensively utilized as a nutritional and immunological supplement for piglets, specifically in modern pig farming, where sows often produce more offspring than they have functional teats for feeding [116]. According to clinical trials with piglets, supplementation with intact BC has been proven to improve piglet survival rates compared with feeding them only with standard formulas, even though it is not as efficient as providing a sow’s colostrum from the mother or a foster mother [117]. Like in human infants, BC enhances immunity in piglets through interactions with gut pathogens and the mucosa. Moreover, combining BC with porcine plasma can increase GI health and even promote development in piglets, demonstrating that BC can act as a partly species-specific substitute for porcine colostrum and can sometimes exceed the benefits of porcine colostrum in terms of gut trophic effects and enzyme maturation [6,112]. Humans have discovered the health benefits of BC in the past and have used BC as an alternative dietary option. Recent studies have explored its use in infant nutrition. A clinical trial performed on preterm pigs indicated that the addition of BC to human milk reduced the risk of gut dysfunction and NEC compared with formula-based fortifiers [118]. In a randomized controlled pilot trial, BC powder in combination with human milk and donor milk was proven to be tolerated by preterm infants, increasing protein intake and plasma tyrosine levels without any significant effects. BC has been suggested as a promising alternative to infant formula because of its potential for promoting intestinal health, enhancing nutrient absorption, and supporting immune defense [119]. It is also advised that standardization of the fortifier composition and the performance of more clinical trials are essential before general use. In addition, compared with traditional bovine milk fortifiers, BC fortifiers have been shown to increase antimicrobial activity in vitro and are considered a viable option for preterm infants [119,120].

## 4. The Potential Roles of Bovine Colostrum in the Context of Pediatric Diseases

### 4.1. Necrotizing Enterocolitis

Necrotizing enterocolitis is a prevalent and severe condition and is the primary cause of GI-related infant deaths, affecting approximately 3–10% of hospitalized preterm infants globally, with a 50% mortality rate [121,122,123]. The disease is the most common GI disease, and the primary victims are premature infants who have managed to survive in the early neonatal periods [124]. NEC was first defined more than a hundred years ago following a set of case studies concerning this disease published between the 1940s and 1950s [125,126]. To date, a sufficient number of clinical and scientific studies have been conducted to elucidate the pathogenetic pathways of NEC, develop therapeutic applications and interventions to prevent NEC disease, and advance the management of this disease [127]. Among the aims of these studies, intervention is the most important factor, and surgical application is required in 20–40% of NEC patients to achieve lower morbidity rates. With respect to the triggering factors of NEC, situations such as the prematurity of infants, the presence of a gut microbiome, and enteral feeding, especially with formulations, are notable. The occurrence of multiple combinations leads to the increased severity of NEC. The essential factor that sufficiently reduces the incidence of NEC in preterm infants is feeding with breast milk, according to current studies. This situation can be explained by the ingredients of HC and milk, which are rich in immune factors [6]. Bovine colostrum, as a substitute for HC, may be a promising source for the intervention of NEC incidence. Some remarkable trials have been conducted to investigate the potential effects of BC in preterm infants.

The results of an open-label randomized controlled trial conducted on 120 preterm infants randomly chosen from either a BC group (n = 60) or a control group (n = 60) revealed that preterm infants in the BC group presented a lower level of feeding intolerance, earlier full enteral intake, a shorter period of parenteral nutrition, and a shorter period of hospital requirement, resulting in high statistical and remarkable significance. The complete results indicated a reduction in NEC development among the BC group. None of the preterm infants in either the BC group or the control group developed severe NEC. Additionally, this study revealed sufficient differences between the two groups in terms of the mean hospitalization period. The mean duration of hospitalization was 23.8 days in the BC group and 31.95 days in the control group. However, the mortality rate between both groups was insignificant, with two deaths (3.3%) in the BC groups and three deaths (5%) in the control group. The findings of this study suggest that the use of bovine colostrum in place of infant formulations during the first week after birth may decrease the incidence of NEC in preterm infants. The time needed to achieve full enteral intake, the period of parenteral nutrition, and the period of hospitalization were also reduced [128]. All of the results of this study may be promising indicators that BC can be exploited and adapted to infant feeding more comprehensively.

Recent studies on the interaction between colostrum and NEC have revealed persistent and expected results in this disease. However, previous studies have indicated that colostrum and NEC are either not correlated or that low levels of correlation are observed. A study by Balachandran et al. (2017) reported no significant differences in the incidence rates of NEC and other related diseases upon the administration of BC compared with a placebo. The study was designed as a blinded, parallel-group, block-randomized, and placebo-controlled trial including infants with a birth weight of 1.5 kg, a gestational age of 32 completed weeks, and a chronological age of 96 h. Notably, there was a limitation in this study, as its sample size was too small to verify the obtained results [129]. In a complete set of studies, it was reported that infants who were administered colostrum were able to establish full enteral feeding earlier than those who were administered a placebo or no intervention. Although early development stemming from colostrum administration was proven clinically in preterm infants, the included studies did not show consistent evidence of an effect on the length of hospitalization [130]. Tao et al. (2020) reviewed related studies regarding the effects of colostrum on a set of diseases, including NEC, via a meta-analysis of randomized controlled trials (RCTs). Nine studies, with a total of 689 preterm infants, compared the incidence of NEC. The NEC incidence rate was determined to be 4.7% in the colostrum group compared with 7.7% in the control group. The pooled results indicated no statistically significant difference between the colostrum group and the control group. The development of NEC in the colostrum group was 41% lower than that in the control group [131]. Similarly, a meta-analysis by Sadeghirad et al. (2018) revealed that eight studies (n = 385) were eligible in demonstrating the prevention of NEC. Compared with a placebo, human or bovine colostrum had no effect on severe NEC infants. The only significant difference was in the reduction in the mean days for full enteral feeds (mean difference: −3.55 days). This analysis revealed that the indirect comparison of BC vs. HC showed no effect on any outcome [132]. Until the 2020s, trials and meta-analyses have shown no direct influence of BC on NEC infants. The most common limitation in these trials was the number of patients, where a modest or insignificant number of subjects hindered certain indications for the use of BC in NEC incidence. Future clinical trials are needed to elucidate the effects of BC more clearly.

### 4.2. Infectious Diarrhea

Diarrhea and related diseases are the main causes of more than half a million deaths in children under 5 years of age worldwide, especially in developing countries [133,134]. In developed countries such as those within Europe and the USA, this disease rarely causes death; nevertheless, it is a significant leading cause of hospitalization and emergency requirements [135]. Acute diarrhea, according to the definition by the World Health Organization (WHO), is the passage of three or more loose or liquid stools per day for 3 or more days and less than 14 days [136]. “Acute diarrhea” or “diarrheal disease” is the preferred definition of this disease in developing countries and the current literature; developed countries define this disease as “acute gastroenteritis”, indicating that the effects and consequences of diarrhea have an impact on the classification and point of view [137]. The dynamics of diarrhea lead to a certain definition in which diarrhea is a gastrointestinal infection caused by certain microorganisms, such as rotavirus, norovirus, *Salmonella*, *E. coli*, and *Campylobacter* [138].

Since the prevalence of acute diarrhea is high worldwide, a sufficient number of clinical trials and studies have been designed to evaluate the interventions and overall effects of this disease. In the context of pediatrics, several studies have focused mostly on BC and other components that are high in immunoglobulins and have positive effects on the symptoms of diarrhea. In a study performed in Guatemala, the effects of BC on 301 Guatemalan child patients (154 BC and 147 placeboes) aged 6–35 months with acute non-bloody diarrhea were studied in a randomized, double-blind, and placebo-controlled trial by using PTM202, a derivative product from BC containing specific immunoglobulins eliminating rotavirus, enterotoxigenic *E. coli*, Shiga toxin-producing *E. coli,* and *Salmonella*. The results revealed no significant difference in the duration of diarrhea between the groups. However, a significant reduction in at least one targeted pathogen in the stool in the treatment group was observed. As a result, the duration of acute diarrhea among urban Guatemalan children with specific pathogens in the stool was shortened by PTM202 over a 3-day course. These results suggest that PTM202 may be an additional therapeutic agent for the intervention of infectious diarrhea in pediatric populations with similar stool pathogen distributions [139]. In a meta-analysis aimed at investigating the protective effects of BC against infectious diarrhea in children, a systematic search was performed via the literature, and among 166 research articles, only 5 were selected for the final analysis. A total of 324 children were analyzed to investigate the effects of BC (or related byproducts) on infectious diarrhea in the context of stool frequency, the incidence rate of diarrhea, and the presence of the pathogen in the stool. Consequently, this systematic review concluded that BC or its byproducts were indeed effective in diminishing the frequency rate of stool, the incidence rate at the end of the intervention, and positive detection of rotavirus, in which approximately 30% of children with diarrhea were rotavirus positive and had E. coli in stool, in comparison with the placebo [140]. All the data were pooled, and the collective results demonstrated that BC was associated with a significant reduction in the frequency of infectious diarrhea in the stool, with a value of approximately 1.42 times per day. BC intervention was also associated with an incidence rate of disease of 71%. The final evaluation revealed that BC and its byproducts have a significant positive effect on reducing the frequency and relieving the symptoms of infectious diarrhea in children [141].

These positive results are promising since children with severe diarrhea generally have recurring and/or persistent diarrhea symptoms and continuous problems in developing countries [142]. The inexpensive and safe conditions of BC make it a superior alternative in such situations. In Egypt, which is considered a developing country, a clinical trial aiming to evaluate the efficiency and tolerability of BC administration for the prevention of recurrent upper respiratory tract infections (URTIs) and diarrhea in 160 children (aged between 1 and 6 years) with previously mentioned diseases was conducted, with BC administered for 4 weeks. The number of episodes of the mentioned diseases and the frequency of hospitalization required for diseases during the study were assessed at weeks 8 and 24. The results indicated that after the administration of BC, the mean (±) SD number of episodes of diarrhea decreased from 6.1 ± 2.0 at baseline to 3.7 ± 2.5 at the end of 2 months. It has also been reported that BC is effective in the prevention of recurrent URTIs and diarrhea, as it causes a reduction in the number of episodes and hospitalizations. These results suggest that BC could be a novel therapeutic option for children with recurrent URTIs and diarrhea [143]. In general, BC is considered an alternative and curative source for treating GI tract diseases, and diarrhea is one of the most investigated diseases in BC. The limitations of BC use are affected by several factors, including its inability to target specific pathogens, the potential for allergic reactions in infants with milk protein sensitivities (discussed in Section 5), and the lack of a standardized dose for treating diarrhea. Further studies are advised to understand this more clearly.

### 4.3. Inflammatory Bowel Disease

Inflammatory bowel disease (IDB) is a chronic, relapsing inflammatory disease affecting the gastrointestinal compartments. There are two main IDB diseases, namely Crohn’s disease (CD) and ulcerative colitis (UC) [144]. The pathogenesis and causes of IDB are due primarily to genetic and environmental factors, microbiota alterations, and strong immune responses [145,146,147]. The dysregulated immune response in IDB patients is linked to T helper (Th) 1 cells in CD patients and Th2 cells in UC patients [148]. The main mechanism of IDB pathogenesis is the activation of Th17 cells, which release IL-17, and the altered cross-regulation between Th17 and regulatory T cells in the GI tract of IDB patients [149]. If the intestinal epithelium, covered by the mucosal layer and in contact with extrinsic factors such as food antigens and bacteria, is damaged, intestinal inflammation may occur, in which dysfunction of the intestinal epithelium is also associated with nutrient malabsorption [150]. The treatment options for IDB patients include anti-inflammatory drugs (aminosalicylates and corticosteroids), immunosuppressive agents (methotrexate and azathioprine), antibiotics, and biological agents (infliximab and vedolizumab) [147,151,152]. Alternative therapeutic applications, such as a healthy lifestyle, personalized diets, and avoiding stress, are also possible for IDB patients [153,154]. Even if these approaches are used for IDB, they are insufficient, and more effective therapies are essential. In this regard, BC may be a potential therapeutic approach, and several related investigations have validated that BC ingredients may have an impact on the clinical course of the GI tract, including the prevalence of IDB [155].

The ingredients of BC have been investigated for IBD treatment in a few studies. These studies were conducted mainly in vitro and occasionally in mouse models, along with a few human clinical trials. One of the studies by Lee et al. (2019) indicated that both whole and whey BC fractions might suppress lipopolysaccharide-induced NF-κB activation in mouse adipocytes. The anti-inflammatory and antioxidative effects of whole BC were much greater than those of whey BC. In mice with DSS-induced colitis, the administration of BC aided in epithelial regeneration [156]. In the same mouse model, BC supplementation also contributed to clinical recovery from colitis [155]. Current studies on BC supplementation have focused on healthy subjects, primarily athletes and children [157]. Another study on BC revealed a correlation between BC supplementation and cytokine secretion in the peripheral blood mononuclear cells (PBMCs) of four male endurance athletes. The concentration of BC led to the increased secretion of IL-2, IL-10, and IFN-γ [158]. Aside from the indirect studies mentioned, there are currently no direct studies specifically investigating the effects of BC in patients with IDB. The sole study by Khan et al. (2002) was a randomized, double-blind, and controlled trial which examined the efficiency of colostrum enemas in the treatment of distal colitis. Fourteen patients (eight females) with a mean age of 45 years (17–75 years range) and mild to moderate severe distal colitis were administered colostrum enemas (100 mL of a 10% solution) or a placebo (albumin solution) for 4 weeks. The activity of the disease was monitored at 0, 2, and 4 weeks. The study concluded that at the end of the fourth week, the colostrum group presented a mean reduction in symptom scores and disease remission in most patients with active left-sided colitis, whereas patients administered mesalazine with placebo enemas presented minimal levels of recurrence [159]. In the context of pediatric and child health, no clinical trials are currently available, and studies indicate that the activity of IBD has been significantly reduced. Common limitations such as sufficient evidence and potential allergic reactions are also present in IBD trials. Further studies regarding child health and pediatry are necessary to optimize and visualize the potential and proven effects of BC.

### 4.4. Short Bowel Syndrome

Short bowel syndrome (SBS) is a complication of extensive intestinal resection or atresia that commonly occurs in infants and children. This disease may occur in two periods: after birth or in older childhood. In newborns, this complication may arise most commonly from necrotizing enterocolitis (NEC), midgut volvulus, and gastroschisis but more commonly from trauma, intestinal thrombosis, and surgical failure in older children [160]. The progression of the disease can be used to classify SBS patients into two subgroups. According to the functional capability of the remaining gut, the disease is categorized as intestinal insufficiency (II) or intestinal failure (IF). II has been defined as a reduction in the functional gut with preserved ability, resulting in the digestion and absorption of a sufficient amount of nutrients along with fluids from a conventional diet to preserve growth. IF has been defined as a reduction in the functional gut without preserved ability, resulting in the inability to digest and absorb enough nutrients and fluids in a conventional diet, maintaining growth and preventing parenteral nutrition (PN) [161,162,163,164]. It can be concluded that II patients are non-PN-dependent, whereas IF patients are PN-dependent [165].

For other GI tract diseases, the effects of BC in SBS have been investigated. Since the beneficial ingredients and effects of BC are well known, a certain number of animal trials and studies are present in the current literature. A clinical study was conducted to examine the effectiveness of BC in improving intestinal function in children with short bowel syndrome (SBS) through metabolic balance assessments. The study involved nine children with SBS in a randomized crossover design, where 20% of their enteral fluid intake was replaced with either BC or a mixed milk diet for four weeks. Energy and wet weight absorption were measured to evaluate the impact of BC on intestinal function. The results revealed that, compared with a mixed milk diet, a BC diet did not increase energy or wet weight absorption. There were also no significant differences in growth or development, as measured by weight and knemometry, between the two diets. Additionally, less than 150% of enteral energy absorption met the basal metabolic rate, and only 50% of enteral fluid absorption met the basal fluid requirements, indicating intestinal failure and the continued necessity for PN. In summary, the study concluded that a diet supplemented with BC did not improve intestinal functions [165]. Another study, similar to the previous one, hypothesized that minimal enteral nutrition (MEN) with BC would stimulate the adaptation of the intestine, rather than formula, and would be sufficiently tolerated in SBS patients because of the growth factors in BC. In two distinct experiments involving 3-day-old piglets and five infants, BC was added to the determined diets. In experiment 2, the tolerance and feasibility of BC supplementation were monitored in a pilot study with five infants suffering from intestinal resection, and the results were compared with those of five previously resected infants as controls. After experiment 2, it was concluded that enteral BC supplementation was well tolerated, as expected, and that no infants experienced clinical symptoms of cow milk allergy [166]. As mentioned, colostrum was well tolerated by newly resected infants. Nevertheless, the clinical outcomes of BC supplementation in infants subjected to intestinal resection are still unclear and require further study. Even if the number of infant studies is limited compared with that of animal studies (especially piglets), further and more comprehensive studies are necessary for preterm infants to acquire more persistent and certain results and trustworthy data. Some piglet studies have investigated the effects of BC on short bowel disease [166,167,168,169]. To date, all the collected data imply that the efficiency of BC in developing SBS patients is mostly dependent on the maturation rate, along with other related factors [6]. It should be considered that individuals with SBS consult their healthcare providers before administering BC into their diets to ensure optimization for their specific conditions.

### 4.5. Neonatal Sepsis

Neonatal sepsis is a systemic disease that originates from bacteria, viruses, or fungi and is associated with circulatory alterations and other clinical manifestations. Although various definitions yet no consensus exists for neonatal sepsis, this condition is traditionally characterized as the isolation of a pathogen from a sterile body fluid such as blood. These alterations ultimately lead to morbidity and mortality [170]. According to the age of onset and timing of the sepsis episode, neonatal sepsis can be classified as early-onset sepsis (EOS) or late-onset sepsis (LOS). Clinical manifestations of early-onset infections generally emerge within the first 72 h [171]. Early-onset infections usually occur before or during birth and are due to vertical mother-to-infant transmission. However, late-onset infections occur after birth (usually between 3 and 7 days) and are linked to harmful organisms present in the hospital environment or society [170]. Even though the basis for this disease has several distinct factors, the effects on the body and the systemic consequences of neonatal sepsis involve common pathways that may be evaluated during investigations.

According to the current literature, few clinical trials or studies have been conducted on human infants with BC, although some preterm pig studies and related studies are available. In 2009, Manzoni et al. (2009) and colleagues conducted a trial on 11 tertiary neonatal intensive care infants by using BLF, where its peak levels were encountered in day 1 colostrum, to determine whether BLF alone or in combination with LGG reduces the incidence of LOS in a double-blind, placebo-controlled, and randomized trial. The trial concluded that the incidence of LOS was lower in both the BLF and BLF with LGG groups (5.9% and 4.6%, respectively) than in the control group (17.3%). Additionally, bacterial or fungal-originated sepsis levels decreased [172]. BLF or BLF with LGG could reduce the incidence of the first episode of LOS. Similarly, Alanwary Abdel et al., 2022 [128], conducted a series of experiments regarding the beneficial influences of BC on preterm infants. The results reported that the incidence rates of culture-proven LOS groups and clinically suspected LOS groups were not significantly different. Moreover, fewer sepsis episodes were observed in the BC group than in the control group [128]. Undoubtedly, these trials have elucidated the effects of colostrum on LOS, but it should be remembered that there is no direct and appropriate application of colostrum for LOS, and further studies should be designed, performed, and evaluated to extend the collection of data and all aspects of incidence effects. As previously mentioned, some preterm pig studies exist. Studies have reported that BC supplementation provides blood bacterial disinfection and supports hemodynamics, resulting in the prevention of septic shock [173].

### 4.6. Gastrointestinal and Respiratory Infections

Because of premature birth and inappropriate development, preterm infants are susceptible to certain diseases related to harmful microorganisms as well as insufficient systems. One of the most common diseases in these infants is gastrointestinal disease, which causes several types of disease, such as feeding intolerance and short bowel syndrome. Feeding intolerance, which is the most common GI disease in preterm infants, causes the malfunction of the intestines and the inability to digest enteral nutritional intake [174]. For previously examined diseases, BC is also able to promote the maturation of the intestine as well as GI digestion and absorption [175,176,177]. In a recent study, feeding intolerance was reduced in the BC group compared with the control group. The results also indicate that gut maturation involves both intestinal differentiation and proliferation and that these developments might be attributed to growth factors in BC, such as IGF (which has improved effects on feeding tolerance) [178]. All of these results suggest that BC can decrease the incidence of feeding intolerance [174].

In bacterial and pathogenic GI disorders caused by bacterial strains such as *E. coli*, *Staphylococcus aureus*, *Enterobacter aerogenes*, *Salmonella typhi*, *H. pylori*, and *Proteus vulgaris*, BC is mostly and commonly used to control and treat all infections caused by exposure to the preceding strains. Studies investigating various aspects of BC have concluded that the development of bacterial strains such as *E. coli*, *S. aureus*, *P. vulgaris*, *E. aerogenes*, and *S. typhi* has been hindered by BC, and the adult Wistar rats used in a particular study presented antimicrobial resistance [114]. Among all these bacteria, the most common species is *H. pylori*, which causes the most prevalent bacterial gastrointestinal infections in humans worldwide. The systemic intake of this bacterium is mostly due to oral entry, resulting in GI infections from the stomach to the later compartments. Owing to the viability of this bacterium in a highly acidic stomach environment, a series of diseases, namely, chronic gastritis, peptic and duodenal ulcers, and gastric adenocarcinoma, occur in the stomach [179]. Since the target of *H. pylori* is the receptor found in the mucosal layer of the stomach, the interaction between these receptors and bacteria was investigated in an in vitro study using BC concentrate (BCC) as an intervention agent. The study concluded that BCC was able to hinder the microbial adhesion of *H. pylori* bacteria to the lipid receptors of the mucosal layer, namely, gangliotetraosylceramide (Gg4), gangliotriaosylceramide (Gg3), and phosphatidylethanolamine (PE). The study also included *Helicobacter mustelae* strains, with similar results [180]. In addition, related studies on animal models regarding the effects of BC on *Helicobacter* strains have shown that BC and its components (i.e., lactoferrin and immunoglobulins) are capable of hindering bacterial viability and binding to receptors along with their antigens [181,182,183,184]. BLF was examined in this respect. Megahed et al. (2017) designed an experiment in which 50 patients were selected and divided into two groups; namely, group 1 was treated with traditional therapy (including clarithromycin, omeprazole, amoxicillin, or metronidazole), and group 2 was treated with BLF supplemented with traditional therapy for a 1-week period. Finally, they reported that the elimination of *H. pylori* infection was greater with BLF-supplemented treatment than with traditional treatment (92% and 68%, respectively) [185]. These results emphasize the importance of BC in the treatment of GI diseases caused by bacterial strains and suggest that the specific components of BC can diminish infections or their downstream effects. Undoubtedly, BC contributes to the GI tract by providing prebiotic components to the microbiota [186]. Nevertheless, more specific studies regarding different mechanisms and their interventions by BC components are necessary to comprehend the extent of BC in GI diseases.

### 4.7. Other Conditions

The inclusive and versatile nature of BC results in a rich component pool, and newborns are susceptible to various external factors, such as bacterial infections from several bodily compartments. Owing to the undeveloped systems of the body, specifically digestion and respiration, BC provides the first shield through its components and prepares the body for its first encounter against these external factors. In this context, BC supplementation has been used to recover additional harmful situations for preterm infants. URTIs include the most common tract infections caused by respiratory viruses. Studies regarding URTI and its symptoms indicate that BC can be an effective agent because of its incidence and severity [68]. A clinical study aiming to evaluate the effectiveness and tolerability of BC in the prevention of URTIs was performed in children. For this purpose, 160 children aged 1–6 years with repetitive URTIs were given BC for 4 weeks. According to the results, the mean total number of URTIs decreased radically after BC supplementation (*p* < 0.001). The study also concluded that BC is efficient in the prevention of repetitive URTIs by reducing the number of episodes and the hospitalization time [143]. In URTIs, other studies also claim the same effects for these episodes [187,188].

BC-supported trials have been conducted in the context of cancer and its complications. Childhood leukemia, the most prevalent cancer in children, causes gastrointestinal mucositis, which promotes morbidity and mortality through adverse effects due to cytotoxic anticancer treatment. This effect leads to a disease called chemotherapy-induced mucositis (CIM), which is defined as an inflammatory effect affecting mucosal surfaces and submucosal layers [6]. In an attributed randomized and placebo-controlled clinical study, newly diagnosed children were subjected to BC or placebo administration for 4 weeks. The results indicated that no alterations were observed in fever, infectious morbidity, or inflammatory responses. However, the results and data might reduce the peak severity levels of oral mucositis [189]. Since these studies are limited to patients with chemotherapy-induced GI toxicity, more comprehensive and inclusive studies are needed to verify these data and make these findings more comparable. BC undoubtedly has favorable effects on certain complications of acute lymphoblastic leukemia (ALL) induction treatment, but further trials will ensure the optimization and more accurate effects of BC in CIM.

## 5. Bovine Colostrum and Pediatric Health: Safety Concerns

BC is commonly used and applied in pediatric studies for its beneficial effects on several diseases and deficiencies, but dietary supplements for infants and children must be well examined in terms of short- and long-term safety. The determinants for BC to be used in pediatrics vary widely in terms of product supply, quality, purity, and microbiological safety; harmful microorganisms may develop in BC, resulting in contamination, severe child allergy risks, excessive supplies of components such as proteins and growth factors, a lack of certain BC components, and the inhibition of drug absorption [6]. Unfortunately, these factors have not been investigated sufficiently to interpret the risks of BC in pediatric patients since the current literature only partially mentions the risks of BC in a study-related manner. Bovine milk is the most preferred infant diet alternative worldwide [190]. Unfortunately, the major and most common reasons for food allergy reactions during early childhood are also triggered by milk along with bovine milk [191]. The most studied and defined risk is cow milk allergy, which can be triggered by the presence of IgE, which adversely affects reactions with milk proteins. The subsequent ingestion of these altered proteins might cause sensitivity or allergy. This allergic reaction is the most prevalent condition; therefore, approximately 2–3% of infants suffer from this disease, even in developed countries, which may indicate that developing countries have a greater rate of CMA in infants [19]. The current studies do not indicate any direct correlation between BC and CMA development other than bovine milk or infant formula. However, various milk products, such as bovine colostrum and yogurt, can induce similar allergic reactions due to their common ingredients in comparison with milk [192,193]. In addition to IgG, which is known to be nonallergenic and found only in BC, α-lactalbumin and β-lactoglobulin, which are allergenic factors that trigger allergies in bovine milk, can also be found in BC [6,193,194]. Because all ruminant mammalian milks share homologous proteins with the same structural and biological properties, cross-reactivity is likely possible in individuals and infants with allergies [192]. BC may lead to similar allergic reactions; thus, the prevalence, diagnosis, and treatment of CMA can be followed by milk approaches at this point. However, BC can be considered effective against allergic reactions based on its well-known rich components [29]. More BC-specific studies are essential in the future to evaluate the potential risk of BC in infant feed.

In a general sense, milk allergy is evaluated as a nontreatable disorder. Therefore, CMA can also be classified specifically as nontreatable. After diagnosis, patients with allergic reactions must avoid allergy-inducing food and nutrients to avoid allergic complications. Most of the time, CMA infants can overcome allergic reactions in adulthood, even though 15% of patients continue to suffer throughout adulthood [193]. In addition to this natural tolerance to milk components, there may be a diminishing portion of IgE due to the lack of milk consumption at the beginning or early stages of life [191]. When this tolerance and its symptoms are observed, the diagnosis of CMA can be commenced, followed by in vitro and in vivo tests, and finished with an oral food challenge (OFC) and a double-blind placebo-controlled food challenge (DBPCFC) by a specialist. The in vitro diagnostic test involves the detection of milk allergen-specific IgE in blood serum. After the diagnosis, the results are interpreted, and the OFC test results determine the conclusion. The detailed diagnostic procedure is described in this section [195]. The only treatment for CMA is a tolerable diet, but in the case of accidental consumption, medical applications, such as oral antihistamines for mild reactions or epinephrine for systemic and respiratory reactions, are possible [191,193,196]. If BC causes CMA, the same approach can be followed for the prevalence, diagnosis, and therapy of this disease.

BC has critical physiological and protective functions in neonates, such as immune-boosting, growth-promoting, and antimicrobial properties that aid in tissue development and the maturation of the digestive system and other essential organs in both neonatal mammals and humans. The immunoglobulins and lactoferrin in BC are vital for building natural immunity in neonates, aiding in the reduction in mortality rates in this age group. Importantly, unlike mature milk, BC has a lower lactose content, which makes it a better substitute for lactose intolerance in humans [197]. The composition of BC also includes glycans, which are crucial for immune defense. In the presence of glycans, pathogenic pathogens such as *E. coli*, *S. aureus*, *H. pylori*, rotavirus, and respiratory viruses are inhibited. In addition to providing essential nutrients, HC glycans also aid in the prevention of pathogen adhesion, the modulation of mucosal immune functions, and the support of healthy gut microbiota [88]. Nonetheless, there is limited information on the safety and efficacy of using BC for healthy term infants who do not have access to their own mothers’ milk immediately after birth in cases of adverse conditions such as maternal illness or the inability to breastfeed. In general, such infants are fed infant formula or donor human milk where available [6]. As a result, while BC offers an enormous level of developmental and health-related advantages, there is no strong rationality for using BC as a supplement for healthy-term infants in long-term periods, especially in developed countries.

## 6. Conclusions

In this review, we extensively reviewed colostrum and its effects on pediatric diseases and infant health. BC is an important nutrient source for infants of mammalian species and is secreted immediately after birth. Each component of colostrum offers significant benefits to health by providing essential elements for critical compartments of body systems. It possesses anti-inflammatory, antioxidant, antibacterial, prebiotic, and antiviral effects, which are discussed separately in this review and provide a unique profile for colostrum. Bovine colostrum, which is the most consumed and prevalent colostrum worldwide, is frequently used in pediatric studies because of its shared homology with HC. The results of the composition and clinical studies of human and bovine colostrum show that BC can be the best alternative source for HC in infant feeding. The richer fat and protein ingredients of colostrum compared to milk provide essential immune protection. Remarkable components of colostrum contribute to infant health and regulate their systems. Lactoferrin promotes cellular proliferation and the growth of mutual bacteria, contributes to immune protection, and has potential therapeutic features. The multi-diverse nature of LF has also been investigated solely in dedicated studies. Lysozyme and α-lactalbumin also have protective effects by inhibiting harmful microorganisms and antibacterial effects, respectively. Immunoglobulins are essential for immune protection and prevent bacterial and viral infections as well as the activation of the immune system. The MFGM promotes healthy microbiota and reduces infections and related inflammation. The MFGM also affects neurodevelopment. Glycans, which are highly variable and versatile oligosaccharides, play crucial roles in immune functions and cell interactions. The colostrum contains high levels and variables of these glycans, supporting GI health and immune development in infants. Glycans are consumed by probiotics and shape the gut microbiota, resulting in improvements in the digestive system. However, alterations in glycosylation pathways are attributed to distinct autoimmune diseases, such as Crohn’s disease, where altered glycan structures on antibodies correlate with disease severity. The potential therapeutic benefits of colostrum include the modulation of immune responses and the improvement of glycosylation pathways, although further research is needed. Additionally, milk oligosaccharides have shown promise in protecting against infections and NEC in preterm infants, indicating their importance in infant nutrition and health. All the components of colostrum have potential and determined benefits. Solely or altogether, studies of these components are necessary to optimize their dose-dependent use and compensate for their adverse effects.

These findings indicate that BC has diverse systemic effects in all parts of its life cycle. Considering the evolutionary mechanisms and timing of BC secretion, the primary role of BC is evaluated as an onset for immune promotion rather than nutritional support, since infants possess deficiencies in various systems. Premature birth makes this situation worse since crucial systems are inappropriately developed. To support preterm infants precisely, BC can be exploited solely or completely in infant formulas. It should also be remembered that there is no universally agreed-upon standard dosage for bovine colostrum in infants. Optimization, dose adjustments, and infant parameters must also be assessed to minimize adverse effects. BC has been proven to have beneficial effects on certain infant disorders, such as necrotizing enterocolitis, bowel syndrome, infectious diarrhea, and gastrointestinal tract (GI tract) disorders, by disrupting severe mechanisms, providing insufficient elements, and mediating reactions. However, ineffective or adverse effects of BC have been observed during clinical trials due to its elements, and these elements should be minimized when clinical experiments are designed. Since BC is a supplement and not a trustworthy treatment, its use should be guided by a healthcare professional, particularly in preterm infants or those with medical conditions. Additionally, more clinical studies with more subjects are needed to gather more data and assess the observed all-scale effects comprehensively. Future research on bovine colostrum should focus on determining optimal dosages, formulations, and safety profiles, particularly for allergic reactions in infants with cow’s milk protein allergies or lactose intolerance. Additionally, studies are needed to address quality control and ensure product safety. Long-term studies should investigate the ongoing effects of colostrum supplementation on immune function and gut health in infants. Comparative studies should also be designed and examined to investigate the benefits of BC in relation to HC. Versatile and unique patient profiles also need to be obtained to assess the diversity of BC effects on infant diseases. Ultimately, the use of BC, often in pediatric conditions and neonatal health, is highly recommended.

## Figures and Tables

**Table 1:** The mean (±) concentrations of immunoglobulins in BC and bovine milk are shown. After parturition, the concentration of Igs is at its highest. Consequently, the primary role of milk shifts from protection to nutrition, with mature milk containing significantly lower concentrations of Igs.

Immunoglobulin (g/L)	Bovine Colostrum	Bovine Milk
IgG1	60.5	0.355
IgM	4.9	0.045
IgA	4.7	0.05
IgG2	3.8	0.055

## Abbreviations

BC, bovine colostrum; HC, human colostrum; NEC, necrotizing enterocolitis; IBD, inflammatory bowel disease; LOS, late-onset sepsis; FI, food intolerance; CMA, cow’s milk allergy; Ig, immunoglobulin; LF, lactoferrin; EGF, epidermal growth factor; TGF-β, transforming growth factor β; PDGF, platelet-derived growth factor; IGF, insulin-like growth factor; GI, gastrointestinal; LFR, lactoferrin receptor; BLF, bovine lactoferrin; HLF, human lactoferrin; SARS-CoV-1, severe acute respiratory syndrome coronavirus 1; ROS, reactive oxygen species; MGFM, milk fat globule membrane; FABP fatty acid-binding protein; RNS, reactive nitrogen species; HIV, human immunodeficiency; Glc, glucose; GAG, glycosaminoglycan, HS, heparan sulfate; Gal, galactose; *O*-GalNAc, *O*-linked *N*-acetylgalactosamine; Fuc, fucose; CTLF, *C*-type lectin family; RA, rheumatoid arthritis; IVIg, high-dose intravenous immunoglobulin; HMO, human milk oligosaccharide; SCFA, short-chain fatty acid; DC, dentritic cell; RCT, randomized controlled trial; WHO; World Health Organization; URTI, upper respiratory tract infection; CD, Crohn’s disease; UC, ulcerative colitis; PBMC, peripheral blood mononuclear cell; SBS, short bowel syndrome; II, intestinal insufficiency; IF, intestinal failure; PN, parenteral nutrition; MEN, minimal enteral nutrition; EOS, early-onset sepsis; BCC, bovine colostrum concentrate (BCC); CIM, chemotherapy-induced mucositis; ALL, acute lymphoblastic leukemia; OFC, oral food challenge; DBPCFC, double-blind placebo-controlled food challenge.

## References

[1] Hammon H.M., Liermann W., Frieten D., Koch C. (2020). Review: Importance of colostrum supply and milk feeding intensity on gastrointestinal and systemic development in calves. Animal.

[2] Playford R.J., Weiser M.J. (2021). Bovine Colostrum: Its Constituents and Uses. Nutrients.

[3] Arslan A., Duman H., Kaplan M., Uzkuç H., Bayraktar A., Ertürk M., Alkan M., Frese S.A., Duar R.M., Henrick B.M. (2021). Determining Total Protein and Bioactive Protein Concentrations in Bovine Colostrum. J. Vis. Exp..

[4] Li Y., Juhl S.M., Ye X., Shen R.L., Iyore E.O., Dai Y., Sangild P.T., Greisen G.O. (2017). A Stepwise, Pilot Study of Bovine Colostrum to Supplement the First Enteral Feeding in Preterm Infants (Precolos): Study Protocol and Initial Results. Front. Pediatr..

[5] Goldman A.S. (2002). Evolution of the Mammary Gland Defense System and the Ontogeny of the Immune System. J. Mammary Gland. Biol. Neoplasia.

[6] Sangild P.T., Vonderohe C., Hebib V.M., Burrin D.G. (2021). Potential benefits of bovine colostrum in pediatric nutrition and health. Nutrients.

[7] Karav S., Le Parc A., de Moura Bell J.M.L.N., Frese S.A., Kirmiz N., Block D.E., Barile D., Mills D.A. (2016). Oligosaccharides Released from milk glycoproteins are selective growth substrates for infant-associated bifidobacteria. Appl. Environ. Microbiol..

[8] Blum J., Hammon H. (2000). Colostrum effects on the gastrointestinal tract, and on nutritional, endocrine and metabolic parameters in neonatal calves. Livest. Prod. Sci..

[9] Blum J.W., Hammon H.M. (2000). Bovine colostrum: More than just an immunoglobulin supplier. Schweiz. Arch. Tierheilkd..

[10] Guilloteau P., Le Huërou-Luron I., Chayvialle J.A., Toullec R., Zabielski R., Blum J.W. (1997). Gut Regulatory Peptides in Young Cattle and Sheep. J. Vet. Med. Ser. A.

[11] Korhonen H.J. (2013). Production and properties of health-promoting proteins and peptides from bovine colostrum and milk. Cell. Mol. Biol..

[12] Yalçıntaş Y.M., Baydemir B., Duman H., Eker F., Biçen A.B., Ertürk M., Karav S. (2024). Ay Exploring the impact of colostrum supplementation on athletes: A comprehensive analysis of clinical trials and diverse properties. Front. Immunol..

[13] Bagwe S., Tharappel L.J., Kaur G., Buttar H.S. (2015). Bovine colostrum: An emerging nutraceutical. J. Complement. Integr. Med..

[14] Chae A., Aitchison A., Day A.S., Keenan J.I. (2017). Bovine colostrum demonstrates anti-inflammatory and antibacterial activity in in vitro models of intestinal inflammation and infection. J. Funct. Foods.

[15] Civra A., Francese R., Donalisio M., Tonetto P., Coscia A., Sottemano S., Balestrini R., Faccio A., Cavallarin L., Moro G.E. (2021). Human Colostrum and Derived Extracellular Vesicles Prevent Infection by Human Rotavirus and Respiratory Syncytial Virus in Vitro. J. Hum. Lact..

[16] Stevens E.E., Patrick T.E., Pickler R. (2009). A History of Infant Feeding. J. Périnat. Educ..

[17] Ananthan A., Balasubramanian H., Rao S., Patole S. (2020). Human Milk–Derived Fortifiers Compared with Bovine Milk–Derived Fortifiers in Preterm Infants: A Systematic Review and Meta-Analysis. Adv. Nutr. Int. Rev. J..

[18] Burris A.D., Burris J., Järvinen K.M. (2020). Cow’s Milk Protein Allergy in Term and Preterm Infants: Clinical Manifestations, Immunologic Pathophysiology, and Management Strategies. NeoReviews.

[19] Host A., Halken S. (2014). Cow’s Milk Allergy: Where have we Come from and where are we Going?. Endocrine Metab. Immune Disord.-Drug Targets.

[20] Maffei D., Schanler R.J. (2017). Human milk is the feeding strategy to prevent necrotizing enterocolitis!. Semin. Perinatol..

[21] Michelet M., Schluckebier D., Petit L.-M., Caubet J.-C. (2017). Food protein-induced enterocolitis syndrome—A review of the literature with focus on clinical management. J. Asthma Allergy.

[22] Golkar A., Milani J.M., Vasiljevic T. (2019). Altering allergenicity of cow’s milk by food processing for applications in infant formula. Crit. Rev. Food Sci. Nutr..

[23] McGrath B.A., Fox P.F., McSweeney P.L.H., Kelly A.L. (2016). Composition and properties of bovine colostrum: A review. Dairy Sci. Technol..

[24] Puppel K., Gołębiewski M., Grodkowski G., Slósarz J., Kunowska-Slósarz M., Solarczyk P., Łukasiewicz M., Balcerak M., Przysucha T. (2019). Composition and Factors Affecting Quality of Bovine Colostrum: A Review. Animals.

[25] Ballard O., Morrow A.L. (2013). Human milk composition: Nutrients and bioactive factors. Pediatr. Clin. N. Am..

[26] Bolino M., Duman H., Avcı İ., Kayili H.M., Petereit J., Zundel C., Salih B., Karav S., Frese S.A. (2024). Proteomic and *N*-glycomic comparison of synthetic and bovine whey proteins and their effect on human gut microbiomes. bioRxiv.

[27] West D.W., Mitchell C.J. (2020). Tracking the Fate of Milk Proteins: Better in Whole or in Part?. J. Nutr..

[28] Martin C.R., Ling P.-R., Blackburn G.L. (2016). Review of Infant Feeding: Key Features of Breast Milk and Infant Formula. Nutrients.

[29] Yalçıntaş Y.M., Duman H., López J.M.M., Portocarrero A.C.M., Lombardo M., Khallouki F., Koch W., Bordiga M., El-Seedi H., Raposo A. (2024). Revealing the Potency of Growth Factors in Bovine Colostrum. Nutrients.

[30] Dupont D., Tomé D. (2020). Milk proteins: Digestion and absorption in the gastrointestinal tract. Milk Proteins.

[31] Arslan A., Kaplan M., Duman H., Bayraktar A., Ertürk M., Henrick B.M., Frese S.A., Karav S. (2021). Bovine Colostrum and Its Potential for Human Health and Nutrition. Front. Nutr..

[32] Montagne P., Cuillière M.L., Molé C., Béné M.C., Faure G. (2001). Changes in Lactoferrin and Lysozyme Levels in Human Milk During the First Twelve Weeks of Lactation. Bioactive Components of Human Milk.

[33] Donovan S.M., Odle J. (1994). Growth Factors in Milk as Mediators of Infant Development. Annu. Rev. Nutr..

[34] Eker F., Duman H., Ertürk M., Karav S. (2024). The potential of lactoferrin as antiviral and immune-modulating agent in viral infectious diseases. Front. Immunol..

[35] Kaplan M., Baktıroğlu M., Kalkan A.E., Canbolat A.A., Lombardo M., Raposo A., Alves J.L.d.B., Witkowska A.M., Karav S. (2024). Lactoferrin: A Promising Therapeutic Molecule against Human Papillomavirus. Nutrients.

[36] Gíslason J., Iyer S., Hutchens T., Lönnerdal B. (1993). Lactoferrin receptors in piglet small intestine: Lactoferrin binding properties, ontogeny, and regional distribution in the gastrointestinal tract. J. Nutr. Biochem..

[37] Donovan S.M. (2016). The Role of Lactoferrin in Gastrointestinal and Immune Development and Function: A Preclinical Perspective. J. Pediatr..

[38] Karav S., German J.B., Rouquié C., Le Parc A., Barile D. (2017). Studying lactoferrin *N*-glycosylation. Int. J. Mol. Sci..

[39] Karav S. (2018). Selective deglycosylation of lactoferrin to understand glycans’ contribution to antimicrobial activity of lactoferrin. Cell. Mol. Biol..

[40] Petschow B.W., Talbott R.D., Batema R.P. (1999). Ability of lactoferrin to promote the growth of *Bifidobacterium* spp. in vitro is independent of receptor binding capacity and iron saturation level. J. Med. Microbiol..

[41] Zhang J., Han X., Shan Y., Zhang L., Du M., Liu M., Yi H., Ma Y. (2018). Effect of bovine lactoferrin and human lactoferrin on the proliferative activity of the osteoblast cell line MC3T3-E1 in vitro. J. Dairy Sci..

[42] Embleton N.D., Berrington J.E. (2020). Clinical Trials of Lactoferrin in the Newborn: Effects on Infection and the Gut Microbiome. Milk, Mucosal Immunity and the Microbiome: Impact on the Neonate.

[43] Dall’Agnola A., Tomé D., Kaufman D.A., Tavella E., Pieretto M., Messina A., De Luca D., Bellaiche M., Mosca A., Piloquet H. (2018). Role of Lactoferrin in Neonates and Infants: An Update. Am. J. Perinatol..

[44] Pammi M., Gautham K.S. (2020). Enteral lactoferrin supplementation for prevention of sepsis and necrotizing enterocolitis in preterm infants. Cochrane Database Syst. Rev..

[45] Bolat E., Eker F., Kaplan M., Duman H., Arslan A., Saritaş S., Şahutoğlu A.S., Karav S. (2022). Lactoferrin for COVID-19 prevention, treatment, and recovery. Front. Nutr..

[46] Eker F., Bolat E., Pekdemir B., Duman H., Karav S. (2023). Lactoferrin: Neuroprotection against Parkinson’s disease and secondary molecule for potential treatment. Front. Aging Neurosci..

[47] Brandtzaeg P., Mann W.V. (1964). A Comparative Study of the Lysozyme Activity of Human Gingival Pocket Fluid, Serum, and Saliva. Acta Odontol. Scand..

[48] Ibrahim H.R., Kato A., Kobayashi K. (1991). Antimicrobial effects of lysozyme against gram-negative bacteria due to covalent binding of palmitic acid. J. Agric. Food Chem..

[49] Ferraboschi P., Ciceri S., Grisenti P. (2021). Applications of Lysozyme, an Innate Immune Defense Factor, as an Alternative Antibiotic. Antibiotics.

[50] Morrin S.T., Buck R.H., Farrow M., Hickey R.M. (2021). Milk-derived anti-infectives and their potential to combat bacterial and viral infection. J. Funct. Foods.

[51] Krissansen G.W. (2007). Emerging Health Properties of Whey Proteins and Their Clinical Implications. J. Am. Coll. Nutr..

[52] Eker F., Akdaşçi E., Duman H., Yalçıntaş Y.M., Canbolat A.A., Kalkan A.E., Karav S., Šamec D. (2024). Antimicrobial Properties of Colostrum and Milk. Antibiotics.

[53] Lajnaf R., Gharsallah H., Jridi M., Attia H., Ayadi M. (2020). Antioxidant and antibacterial activities, interfacial and emulsifying properties of the apo and holo forms of purified camel and bovine α-lactalbumin. Int. J. Biol. Macromol..

[54] Diao M., Yan M., Wang Y., Yan X., Dong S., Lu Y., Zhang T. (2022). Characterization and antibacterial activity study of α-Lactalbumin-carvacrol complex. Food Chem..

[55] Ahmad S., Anjum F.M., Huma N., Sameen A., Zahoor T. (2013). Composition and Physico-chemical Characteristics of Buffalo Milk with Particular Emphasis on Lipids, Proteins, Minerals, Enzymes and Vitamins. J. Anim. Plant Sci..

[56] Barrington G., Besser T., Davis W., Gay C., Reeves J., McFadden T. (1997). Expression of Immunoglobulin G1 Receptors by Bovine Mammary Epithelial Cells and Mammary Leukocytes. J. Dairy Sci..

[57] Jensen A.R., Elnif J., Sangild P.T., Burrin D.G. (2001). Development of Intestinal Immunoglobulin Absorption and Enzyme Activities in Neonatal Pigs Is Diet Dependent. J. Nutr..

[58] Sangild P. (2003). Uptake of Colostral Immunoglobulins by the Compromised Newborn Farm Animal. Acta Vet. Scand..

[59] Marnila P., Korhonen H. (2011). Milk|Colostrum. Encyclopedia of Dairy Sciences.

[60] Rajala P., Castrén H. (1995). Serum Immunoglobulin Concentrations and Health of Dairy Calves in Two Management Systems from Birth to 12 Weeks of Age. J. Dairy Sci..

[61] Baumrucker C.R., Bruckmaier R.M. (2014). Colostrogenesis: IgG1 Transcytosis Mechanisms. J. Mammary Gland. Biol. Neoplasia.

[62] Kaskous S., Fadlelmoula A.A., Kaskous S., Fadlelmoula A. (2015). Immunoglobulin in Colostrum and Health of Newborn Calves (Review) Immunoglobulin in colostrum and health of newborn Calves. Sci. J. Rev..

[63] Lee H., Padhi E., Hasegawa Y., Larke J., Parenti M., Wang A., Hernell O., Lönnerdal B., Slupsky C. (2018). Compositional Dynamics of the Milk Fat Globule and Its Role in Infant Development. Front. Pediatr..

[64] Koletzko B. (2016). Human Milk Lipids. Ann. Nutr. Metab..

[65] Manoni M., Di Lorenzo C., Ottoboni M., Tretola M., Pinotti L. (2020). Comparative Proteomics of Milk Fat Globule Membrane (MFGM) Proteome across Species and Lactation Stages and the Potentials of MFGM Fractions in Infant Formula Preparation. Foods.

[66] Bhinder G., Allaire J.M., Garcia C., Lau J.T., Chan J.M., Ryz N.R., Bosman E.S., Graef F.A., Crowley S.M., Celiberto L.S. (2017). Milk Fat Globule Membrane Supplementation in Formula Modulates the Neonatal Gut Microbiome and Normalizes Intestinal Development. Sci. Rep..

[67] Salcedo J., Karav S., Le Parc A., Cohen J.L., de Moura Bell J.M., Sun A., Lange M.C., Barile D. (2018). Application of industrial treatments to donor human milk: Influence of pasteurization treatments, storage temperature, and time on human milk gangliosides. npj Sci. Food.

[68] Duman H., Karav S. (2023). Bovine colostrum and its potential contributions for treatment and prevention of COVID-19. Front. Immunol..

[69] Hernell O., Lönnerdal B., Timby N. (2020). Milk Fat Globule Membranes: Effects on Microbiome, Metabolome, and Infections in Infants and Children. Milk, Mucosal Immunity and the Microbiome: Impact on the Neonate.

[70] Honan M.C., Fahey M.J., Fischer-Tlustos A.J., Steele M.A., Greenwood S.L. (2020). Shifts in the Holstein dairy cow milk fat globule membrane proteome that occur during the first week of lactation are affected by parity. J. Anim. Sci. Biotechnol..

[71] Tanaka K., Hosozawa M., Kudo N., Yoshikawa N., Hisata K., Shoji H., Shinohara K., Shimizu T. (2013). The pilot study: Sphingomyelin-fortified milk has a positive association with the neurobehavioural development of very low birth weight infants during infancy, randomized control trial. Brain Dev..

[72] Palmano K., Rowan A., Guillermo R., Guan J., Mc Jarrow P. (2015). The Role of Gangliosides in Neurodevelopment. Nutrients.

[73] Rueda R., Maldonado J., Narbona E., Gil A. (1998). Neonatal dietary gangliosides. Early Hum. Dev..

[74] Hernell O., Timby N., Domellöf M., Lönnerdal B. (2016). Clinical Benefits of Milk Fat Globule Membranes for Infants and Children. J. Pediatr..

[75] Spitsberg V. (2005). Invited Review: Bovine Milk Fat Globule Membrane as a Potential Nutraceutical. J. Dairy Sci..

[76] Kaplan M., Şahutoğlu A.S., Sarıtaş S., Duman H., Arslan A., Pekdemir B., Karav S. (2022). Role of milk glycome in prevention, treatment, and recovery of COVID-19. Front. Nutr..

[77] Abeler-Dörner L., Swamy M., Williams G., Hayday A.C., Bas A. (2012). Butyrophilins: An emerging family of immune regulators. Trends Immunol..

[78] Arnett H.A., Viney J.L. (2014). Immune modulation by butyrophilins. Nat. Rev. Immunol..

[79] Timby N., Domellöf M., Holgerson P.L., West C.E., Lönnerdal B., Hernell O., Johansson I. (2017). Oral Microbiota in Infants Fed a Formula Supplemented with Bovine Milk Fat Globule Membranes—A Randomized Controlled Trial. PLoS ONE.

[80] Urashima T., Taufik E., Fukuda K., Asakuma S. (2013). Recent Advances in Studies on Milk Oligosaccharides of Cows and Other Domestic Farm Animals. Biosci. Biotechnol. Biochem..

[81] Duman H., Kaplan M., Arslan A., Sahutoglu A.S., Kayili H.M., Frese S.A., Karav S. (2021). Potential Applications of Endo-β-*N*-Acetylglucosaminidases from *Bifidobacterium longum* Subspecies *infantis* in Designing Value-Added, Next-Generation Infant Formulas. Front. Nutr..

[82] Albrecht S., Lane J.A., Mariño K., Al Busadah K.A., Carrington S.D., Hickey R.M., Rudd P.M. (2014). A comparative study of free oligosaccharides in the milk of domestic animals. Br. J. Nutr..

[83] Gopal P.K., Gill H.S. (2000). Oligosaccharides and glycoconjugates in bovine milk and colostrum. Br. J. Nutr..

[84] Tao N., DePeters E., Freeman S., German J., Grimm R., Lebrilla C. (2008). Bovine Milk Glycome. J. Dairy Sci..

[85] Varki A. (2017). Biological roles of glycans. Glycobiology.

[86] Varki A., Cummings R.D., Esko J.D., Stanley P., Hart G.W., Aebi M., Mohnen D., Kinoshita T., Packer N.H., Prestegard J.J. (2015). Essentials of Glycobiology.

[87] Zhou J.Y., Cobb B.A. (2021). Glycans in Immunologic Health and Disease. Annu. Rev. Immunol..

[88] Newburg D.S. (2009). Neonatal protection by an innate immune system of human milk consisting of oligosaccharides and glycans1. J. Anim. Sci..

[89] Bunyatratchata A., Le Parc A., de Moura J.M., Cohen J.L., Duman H., Arslan A., Kaplan M., Barile D., Karav S. (2023). Release of bifidogenic *N*-glycans from native bovine colostrum proteins by an endo-β-*N*-acetylglucosaminidase. Enzym. Microb. Technol..

[90] Pekdemir B., Karav S. (2024). Exploring the diverse biological significance and roles of fucosylated oligosaccharides. Front. Mol. Biosci..

[91] Parekh R.B., Dwek R.A., Sutton B.J., Fernandes D.L., Leung A., Stanworth D., Rademacher T.W., Mizuochi T., Taniguchi T., Matsuta K. (1985). Association of rheumatoid arthritis and primary osteoarthritis with changes in the glycosylation pattern of total serum IgG. Nature.

[92] Pekelharing J.M., Hepp E., Kamerling J.P., Gerwig G.J., Leijnse B. (1988). Alterations in carbohydrate composition of serum IgG from patients with rheumatoid arthritis and from pregnant women. Ann. Rheum. Dis..

[93] van de Geijn F.E., Wuhrer M., Selman M.H., Willemsen S.P., de Man Y.A., Deelder A.M., Hazes J.M., Dolhain R.J. (2009). Immunoglobulin G galactosylation and sialylation are associated with pregnancy-induced improvement of rheumatoid arthritis and the postpartum flare: Results from a large prospective cohort study. Arthritis Res. Ther..

[94] Konno N., Sugimoto M., Takagi T., Furuya M., Asano T., Sato S., Kobayashi H., Migita K., Miura Y., Aihara T. (2018). Changes in *N*-glycans of IgG4 and its relationship with the existence of hypocomplementemia and individual organ involvement in patients with IgG4-related disease. PLoS ONE.

[95] Zhang J., Zhao L., Gao Y., Liu M., Li T., Huang Y., Lu G., Gao Y., Guo X., Shi B. (2014). A Classification of Hashimoto’s Thyroiditis Based on Immunohistochemistry for IgG4 and IgG. Thyroid^®^.

[96] Šimurina M., de Haan N., Vučković F., Kennedy N.A., Štambuk J., Falck D., Trbojević-Akmačić I., Clerc F., Razdorov G., Khon A. (2018). Glycosylation of Immunoglobulin G Associates With Clinical Features of Inflammatory Bowel Diseases. Gastroenterology.

[97] Mestecky J., Tomana M., Moldoveanu Z., Julian B., Suzuki H., Matousovic K., Renfrow M., Novak L., Wyatt R., Novak J. (2008). Role of Aberrant Glycosylation of IgA1 Molecules in the Pathogenesis of IgA Nephropathy. Kidney Blood Press. Res..

[98] Youinou P., Pennec Y.-L., Casburn-Budd R., Dueymes M., Letoux G., Lamour A. (1992). Galactose terminating oligosaccharides of IgG in patients with primary Sjögren’s syndrome. J. Autoimmun..

[99] Shade K.-T.C., Conroy M.E., Washburn N., Kitaoka M., Huynh D.J., Laprise E., Patil S.U., Shreffler W.G., Anthony R.M. (2020). Sialylation of immunoglobulin E is a determinant of allergic pathogenicity. Nature.

[100] Cobb B.A. (2020). The history of IgG glycosylation and where we are now. Glycobiology.

[101] Anthony R.M., Wermeling F., Ravetch J.V. (2012). Novel roles for the IgG Fc glycan. Ann. N. Y. Acad. Sci..

[102] Kaneko Y., Nimmerjahn F., Ravetch J.V. (2006). Anti-Inflammatory Activity of Immunoglobulin G Resulting from Fc Sialylation. Science.

[103] Maverakis E., Kim K., Shimoda M., Gershwin M.E., Patel F., Wilken R., Raychaudhuri S., Ruhaak L.R., Lebrilla C.B. (2015). Glycans in the immune system and The Altered Glycan Theory of Autoimmunity: A critical review. J. Autoimmun..

[104] Oswald D.M., Sim E.S., Baker C., Farhan O., Debanne S.M., Morris N.J., Rodriguez B.G., Jones M.B., Cobb B.A. (2019). Plasma glycomics predict cardiovascular disease in patients with ART-controlled HIV infections. FASEB J..

[105] Karav S., Mills D.A. (2015). Selective Prebiotic Activity of *N*-Glycans Released from Milk Glycoproteins by Novel En-do-β-*N*-acetylglucosaminidase. United States—California. https://www.proquest.com/dissertations-theses/selective-prebiotic-activity-i-n-glycans-released/docview/1774020008/se-2?accountid=15572.

[106] Karav S., Bell J.M.L.N.D.M., Le Parc A., Liu Y., Mills D.A., Block D.E., Barile D. (2015). Characterizing the release of bioactive *N*-glycans from dairy products by a novel endo-β-*N*-acetylglucosaminidase. Biotechnol. Prog..

[107] Lane J.A., Mariño K., Naughton J., Kavanaugh D., Clyne M., Carrington S.D., Hickey R.M. (2012). Anti-infective bovine colostrum oligosaccharides: Campylobacter jejuni as a case study. Int. J. Food Microbiol..

[108] Bode L. (2018). Human Milk Oligosaccharides in the Prevention of Necrotizing Enterocolitis: A Journey From in vitro and in vivo Models to Mother-Infant Cohort Studies. Front. Pediatr..

[109] Sarıtaş S., Portocarrero A.C.M., López J.M.M., Lombardo M., Koch W., Raposo A., El-Seedi H.R., Alves J.L.d.B., Esatbeyoglu T., Karav S. (2024). The Impact of Fermentation on the Antioxidant Activity of Food Products. Molecules.

[110] Ahern G.J., Hennessy A., Ryan C.A., Ross R.P., Stanton C. (2019). Advances in Infant Formula Science. Annu. Rev. Food Sci. Technol..

[111] Bagwe-Parab S., Yadav P., Kaur G., Tuli H.S., Buttar H.S. (2020). Therapeutic Applications of Human and Bovine Colostrum in the Treatment of Gastrointestinal Diseases and Distinctive Cancer Types: The Current Evidence. Front. Pharmacol..

[112] Hurley W.L., Theil P.K. (2011). Perspectives on Immunoglobulins in Colostrum and Milk. Nutrients.

[113] Ulfman L.H., Leusen J.H.W., Savelkoul H.F.J., Warner J.O., van Neerven R.J.J. (2018). Effects of Bovine Immunoglobulins on Immune Function, Allergy, and Infection. Front. Nutr..

[114] Gomes R.D., Anaya K., Galdino A.B., Oliveira J.P., Gama M.A., Medeiros C.A., Gavioli E.C., Porto A.L.F., Rangel A.H. (2021). Bovine colostrum: A source of bioactive compounds for prevention and treatment of gastrointestinal disorders. NFS J..

[115] Kaplan M., Arslan A., Duman H., Karyelioğlu M., Baydemir B., Günar B.B., Alkan M., Bayraktar A., Tosun H.I., Ertürk M. (2022). Production of Bovine Colostrum for Human Consumption to Improve Health. Front. Pharmacol..

[116] Kirkden R.D., Broom D.M., Andersen I.L. (2013). INVITED REVIEW: Piglet mortality: Management solutions. J. Anim. Sci..

[117] Gomez G.G., Phillips O., A Goforth R. (1998). Effect of immunoglobulin source on survival, growth, and hematological and immunological variables in pigs. J. Anim. Sci..

[118] Juhl S.M., Ye X., Zhou P., Li Y., Iyore E.O., Zhang L., Jiang P., van Goudoever J.B., Greisen G., Sangild P.T. (2018). Bovine Colostrum for Preterm Infants in the First Days of Life. J. Pediatr. Gastroenterol. Nutr..

[119] Mehra R., Garhwal R., Sangwan K., Guiné R.P.F., Lemos E.T., Buttar H.S., Visen P.K.S., Kumar N., Bhardwaj A., Kumar H. (2022). Insights into the Research Trends on Bovine Colostrum: Beneficial Health Perspectives with Special Reference to Manufacturing of Functional Foods and Feed Supplements. Nutrients.

[120] Gao X., Li Y., Olin A.B., Nguyen D.N. (2021). Fortification with Bovine Colostrum Enhances Antibacterial Activity of Human Milk. J. Parenter. Enter. Nutr..

[121] Burrin D., Sangild P.T., Stoll B., Thymann T., Buddington R., Marini J., Olutoye O., Shulman R.J. (2020). Translational Advances in Pediatric Nutrition and Gastroenterology: New Insights from Pig Models. Annu. Rev. Anim. Biosci..

[122] Neu J., Walker W.A. (2011). Necrotizing Enterocolitis. N. Engl. J. Med..

[123] Niño D.F., Sodhi C.P., Hackam D.J. (2016). Necrotizing enterocolitis: New insights into pathogenesis and mechanisms. Nat. Rev. Gastroenterol. Hepatol..

[124] Lin P.W., Stoll B.J. (2006). Necrotising enterocolitis. Lancet.

[125] Schmid K.O. (1952). A specially severe form of enteritis in newborn, enterocolitis ulcerosa necroticans. I. Pathological anatomy. Osterr Z Kinderheilkd Kinderfuersorge.

[126] Stiennon O.A. (1951). Pneumatosis intestinalis in the newborn. AMA Am. J. Dis. Child..

[127] Duchon J., Barbian M.E., Denning P.W. (2021). Necrotizing Enterocolitis. Clin. Perinatol..

[128] Abdel S.A., Monem S.A.A., Ezzeldin Z.M., Said H.M., Baris S.S.H., El Tatawy S.S. (2022). Bovine Colostrum Supplementation for Prevention of Necrotizing Enterocolitis and Late-Onset Sepsis in Preterm Infants. NeuroQuantology.

[129] Balachandran B., Dutta S., Singh R., Prasad R., Kumar P. (2017). Bovine colostrum in prevention of necrotizing enterocolitis and sepsis in very low birth weight neonates: A randomized, double-blind, placebo-controlled pilot trial. J. Trop. Pediatr..

[130] Nasuf A.W.A., Ojha S., Dorling J. (2018). Oropharyngeal colostrum in preventing mortality and morbidity in preterm infants. Cochrane Database Syst. Rev..

[131] Tao J., Mao J., Yang J., Su Y. (2020). Effects of oropharyngeal administration of colostrum on the incidence of necrotizing enterocolitis, late-onset sepsis, and death in preterm infants: A meta-analysis of RCTs. Eur. J. Clin. Nutr..

[132] Sadeghirad B., Morgan R.L., Zeraatkar D., Zea A.M., Couban R., Johnston B.C., Florez I.D. (2018). Human and Bovine Colostrum for Prevention of Necrotizing Enterocolitis: A Meta-analysis. Pediatrics.

[133] Black R.E., Morris S., Bryce J. (2003). Where and why are 10 million children dying every year?. Lancet.

[134] Liu L., Oza S., Hogan D., Perin J., Rudan I., Lawn J.E., Cousens S., Mathers C., Black R.E. (2015). Global, regional, and national causes of child mortality in 2000–13, with projections to inform post-2015 priorities: An updated systematic analysis. Lancet.

[135] Schnadower D., Finkelstein Y., Freedman S.B. (2015). Ondansetron and probiotics in the management of pediatric acute gastroenteritis in developed countries. Curr. Opin. Gastroenterol..

[136] World Health Organization (2005). The Treatment of Diarrhoea A Manual for Physicians and Other Senior Health Workers (4th Rev.).

[137] Florez I.D., Niño-Serna L.F., Beltrán-Arroyave C.P. (2020). Acute Infectious Diarrhea and Gastroenteritis in Children. Curr. Infect. Dis. Rep..

[138] Guerrant R.L., Hughes J.M., Lima N.L., Crane J. (1990). Diarrhea in Developed and Developing Countries: Magnitude, Special Settings, and Etiologies. Clin. Infect. Dis..

[139] Gaensbauer J.T., Melgar M.A., Calvimontes D.M., Lamb M.M., Asturias E.J., Contreras-Roldan I.L., Dominguez S.R., Robinson C.C., Berman S. (2017). Efficacy of a bovine colostrum and egg-based intervention in acute childhood diarrhoea in Guatemala: A randomised, double-blind, placebo-controlled trial. BMJ Glob. Health.

[140] Yu J., Lai S., Geng Q., Ye C., Zhang Z., Zheng Y., Wang L., Duan Z., Zhang J., Wu S. (2019). Prevalence of rotavirus and rapid changes in circulating rotavirus strains among children with acute diarrhea in China, 2009–2015. J. Infect..

[141] Li J., Xu Y.-W., Jiang J.-J., Song Q.-K. (2019). Bovine colostrum and product intervention associated with relief of childhood infectious diarrhea. Sci. Rep..

[142] Chandwe K., Kelly P. (2021). Colostrum Therapy for Human Gastrointestinal Health and Disease. Nutrients.

[143] Saad K., Abo-Elela M.G.M., El-Baseer K.A.A., Ahmed A.E., Ahmad F.-A., Tawfeek M.S.K., Houfey A.A.E., Aboul_Khair M.D., Abdel-Salam A.M., Abo-Elgheit A. (2016). Effects of bovine colostrum on recurrent respiratory tract infections and diarrhea in children. Medicine.

[144] Holtmann G.J., Ford A.C., Talley N.J. (2016). Pathophysiology of irritable bowel syndrome. Lancet Gastroenterol. Hepatol..

[145] Bernstein C.N., Eliakim A., Fedail S., Fried M., Gearry R., Goh K.-L., Hamid S., Khan A.G., Khalif I., Ng S.C. (2016). World Gastroenterology Organisation Global Guidelines Inflammatory Bowel Disease. J. Clin. Gastroenterol..

[146] Xu H., Liu M., Cao J., Li X., Fan D., Xia Y., Lu X., Li J., Ju D., Zhao H. (2019). The Dynamic Interplay between the Gut Microbiota and Autoimmune Diseases. J. Immunol. Res..

[147] Zhang Y.-Z., Li Y.-Y. (2014). Inflammatory bowel disease: Pathogenesis. World J. Gastroenterol..

[148] Brand S. (2009). Crohn’s disease: Th1, Th17 or both? The change of a paradigm: New immunological and genetic insights implicate Th17 cells in the pathogenesis of Crohn’s disease. Gut.

[149] Geremia A., Jewell D.P. (2012). The IL-23/IL-17 pathway in inflammatory bowel disease. Expert Rev. Gastroenterol. Hepatol..

[150] Salim S.Y., Söderholm J.D. (2011). Importance of disrupted intestinal barrier in inflammatory bowel diseases. Inflamm. Bowel Dis..

[151] Fichna J. (2016). Inflammatory bowel disease treatment. Pharmacol. Rep..

[152] Yamamoto-Furusho J. (2012). Tratamiento de la enfermedad inflamatoria intestinal. Rev. Gastroenterol. Mex..

[153] Lee D., Albenberg L., Compher C., Baldassano R., Piccoli D., Lewis J.D., Wu G.D. (2015). Diet in the Pathogenesis and Treatment of Inflammatory Bowel Diseases. Gastroenterology.

[154] Sigall-Boneh R., Levine A., Lomer M., Wierdsma N., Allan P., Fiorino G., Gatti S., Jonkers D., Kierkuś J., Katsanos K.H. (2017). Research Gaps in Diet and Nutrition in Inflammatory Bowel Disease. A Topical Review by D-ECCO Working Group [Dietitians of ECCO]. J. Crohn’s Colitis.

[155] Bodammer P., Maletzki C., Waitz G., Emmrich J. (2011). Prophylactic Application of Bovine Colostrum Ameliorates Murine Colitis via Induction of Immunoregulatory Cells. J. Nutr..

[156] Lee A., Pontin M.C., Kosmerl E., Jimenez-Flores R., Moretti D.B., Ziouzenkova O. (2019). Assessment of adipogenic, antioxidant, and anti-inflammatory properties of whole and whey bovine colostrum. J. Dairy Sci..

[157] Sienkiewicz M., Szymańska P., Fichna J. (2021). Supplementation of Bovine Colostrum in Inflammatory Bowel Disease: Benefits and Contraindications. Adv. Nutr. Int. Rev. J..

[158] Shing C.M., Peake J.M., Suzuki K., Jenkins D.G., Coombes J.S. (2009). Bovine Colostrum Modulates Cytokine Production in Human Peripheral Blood Mononuclear Cells Stimulated with Lipopolysaccharide and Phytohemagglutinin. J. Interf. Cytokine Res..

[159] Khan Z., Macdonald C., Wicks A.C., Holt M.P., Floyd D., Ghosh S., Wright N.A., Playford R.J. (2002). Use of the ‘nutriceutical’, bovine colostrum, for the treatment of distal colitis: Results from an initial study. Aliment. Pharmacol. Ther..

[160] Sigalet D. (2001). Short Bowel Syndrome in Infants and Children: An Overview. Semin. Pediatr. Surg..

[161] Goulet O., Ruemmele F., Lacaille F., Colomb V. (2004). Irreversible Intestinal Failure. J. Pediatr. Gastroenterol. Nutr..

[162] Goulet O., Ruemmele F. (2006). Causes and Management of Intestinal Failure in Children. Gastroenterology.

[163] Jeppesen P.B., Mortensen P.B. (2000). Intestinal failure defined by measurements of intestinal energy and wet weight absorption. Gut.

[164] O’keefe S.J., Buchman A.L., Fishbein T.M., Jeejeebhoy K.N., Jeppesen P.B., Shaffer J. (2006). Short Bowel Syndrome and Intestinal Failure: Consensus Definitions and Overview. Clin. Gastroenterol. Hepatol..

[165] Aunsholt L., Jeppesen P.B., Lund P., Sangild P.T., Ifaoui I.B.R., Qvist N., Husby S. (2014). Bovine colostrum to children with short bowel syndrome: A randomized, double-blind, crossover pilot study. J. Parenter. Enter. Nutr..

[166] Aunsholt L., Qvist N., Sangild P.T., Vegge A., Stoll B., Burrin D.G., Jeppesen P.B., Eriksen T., Husby S., Thymann T. (2018). Minimal Enteral Nutrition to Improve Adaptation After Intestinal Resection in Piglets and Infants. J. Parenter. Enter. Nutr..

[167] Aunsholt L., Thymann T., Qvist N., Sigalet D., Husby S., Sangild P.T. (2015). Prematurity Reduces Functional Adaptation to Intestinal Resection in Piglets. J. Parenter. Enter. Nutr..

[168] Pereira-Fantini P.M., Thomas S.L., Taylor R.G., Nagy E., Sourial M., Fuller P.J., Bines J.E. (2008). Colostrum Supplementation Restores Insulin-like Growth Factor -1 Levels and Alters Muscle Morphology Following Massive Small Bowel Resection. J. Parenter. Enter. Nutr..

[169] Sangild P.T., Ney D.M., Sigalet D.L., Vegge A., Burrin D. (2014). Animal models of gastrointestinal and liver diseases. Animal models of infant short bowel syndrome: Translational relevance and challenges. Am. J. Physiol. Liver Physiol..

[170] Shane A.L., Sánchez P.J., Stoll B.J. (2017). Neonatal sepsis. Lancet.

[171] Wynn J.L., Wong H.R., Shanley T.P., Bizzarro M.J., Saiman L., Polin R.A. (2014). Time for a Neonatal-Specific Consensus Definition for Sepsis. Pediatr. Crit. Care Med..

[172] Manzoni P. (2009). Bovine Lactoferrin Supplementation for Prevention of Late-Onset Sepsis in Very Low-Birth-Weight NeonatesA Randomized Trial. JAMA.

[173] Brunse A., Worsøe P., Pors S.E., Skovgaard K., Sangild P.T. (2019). Oral Supplementation with Bovine Colostrum Prevents Septic Shock and Brain Barrier Disruption During Bloodstream Infection in Preterm Newborn Pigs. Shock.

[174] Luo F., Zhang M., Zhang L., Zhou P. (2023). Nutritional and health effects of bovine colostrum in neonates. Nutr. Rev..

[175] Jensen M.L., Sangild P.T., Lykke M., Schmidt M., Boye M., Jensen B.B., Thymann T. (2013). Similar efficacy of human banked milk and bovine colostrum to decrease incidence of necrotizing enterocolitis in preterm piglets. Am. J. Physiol. Integr. Comp. Physiol..

[176] Li Y., Jensen M.L., Chatterton D.E.W., Jensen B.B., Thymann T., Kvistgaard A.S., Sangild P.T. (2014). Raw bovine milk improves gut responses to feeding relative to infant formula in preterm piglets. Am. J. Physiol. Liver Physiol..

[177] Rasmussen S.O., Martin L., Østergaard M.V., Rudloff S., Li Y., Roggenbuck M., Bering S.B., Sangild P.T. (2016). Bovine colostrum improves neonatal growth, digestive function, and gut immunity relative to donor human milk and infant formula in preterm pigs. Am. J. Physiol. Liver Physiol..

[178] Ismail R.I.H., Awad H.A., Imam S.S., Gad G.I., Aboushady N.M., Abdou R.M., Eissa D.S., Azzam N.T., Barakat M.M., Yassin M.M. (2021). Gut priming with bovine colostrum and T regulatory cells in preterm neonates: A randomized controlled trial. Pediatr. Res..

[179] Kao C.-Y., Sheu B.-S., Wu J.-J. (2016). Helicobacter pylori infection: An overview of bacterial virulence factors and pathogenesis. Biomed. J..

[180] Bitzan M.M., Gold B.D., Philpott D.J., Huesca M., Sherman P.M., Karch H., Lissner R., Lingwood C.A., Karmali M.A. (1998). Inhibition of Helicobacter pylori and Helicobacter mustelae Binding to Lipid Receptors by Bovine Colostrum. J. Infect. Dis..

[181] Baltierra-Uribe S.L., Montañez-Barragán A., Romero-Ramírez H., Klimov-Kravtchenko K., Martínez-Pedro K.I., Sánchez-Salguero E., Camorlinga-Ponce M., Torres J., Santos-Argumedo L. (2021). Colostrum IgA1 antibodies recognize antigens from *Helicobacter pylori* and prevent cytoskeletal changes in human epithelial cells. Eur. J. Immunol..

[182] Casswall T.H., Nilsson H.-O., Björck L., Sjöstedt S., Xu L., Nord C.-E., Borén T., Wadström T., Hammarström L. (2002). Bovine Anti- Helicobacter pylori Antibodies for Oral Immunotherapy. Scand. J. Gastroenterol..

[183] Marnila P., Rokka S., Rehnberg-Laiho L., Karkkainen P., Kosunen T.U., Rautelin H., Hanninen M.-L., Syvaoja E.-L., Korhonen H. (2003). Prevention and Suppression of Helicobacter felis Infection in Mice Using Colostral Preparation with Specific Antibodies. Helicobacter.

[184] Tran C.D., Kritas S., Campbell M.A.F., Huynh H.Q., Lee S.-S., Butler R.N. (2010). Novel combination therapy for the eradication of *Helicobacter pylori* infection in a mouse model. Scand. J. Gastroenterol..

[185] Megahed F., El-Assal M., Dabour A.S., Samy R., Rizk M., Al Adhm S.H. (2017). Lactoferrin as an added therapy in the treatment of Helicobacter pylori. Benha Med. J..

[186] Yalçıntaş Y.M., Duman H., Rocha J.M., Bartkiene E., Karav S., Ozogul F. (2024). Role of bovine colostrum against various diseases. Food Biosci..

[187] Brinkworth G.D., Buckley J.D. (2003). Concentrated bovine colostrum protein supplementation reduces the incidence of self-reported symptoms of upper respiratory tract infection in adult males. Eur. J. Nutr..

[188] Patel K., Rana R. (2006). Pedimune in recurrent respiratory infection and diarrhoea—The Indian experience—The PRIDE study. Indian J. Pediatr..

[189] Rathe M., De Pietri S., Wehner P.S., Frandsen T.L., Grell K., Schmiegelow K., Sangild P.T., Husby S., Müller K. (2020). Bovine Colostrum Against Chemotherapy-Induced Gastrointestinal Toxicity in Children with Acute Lymphoblastic Leukemia: A Randomized, Double-Blind, Placebo-Controlled Trial. J. Parenter. Enter. Nutr..

[190] Do A.B., Williams K., Toomer O.T. (2016). In vitro digestibility and immunoreactivity of bovine milk proteins. Food Chem..

[191] Hochwallner H., Schulmeister U., Swoboda I., Spitzauer S., Valenta R. (2014). Cow’s milk allergy: From allergens to new forms of diagnosis, therapy and prevention. Methods.

[192] Monaci L., Tregoat V., Van Hengel A.J., Anklam E. (2006). Milk allergens, their characteristics and their detection in food: A review. Eur. Food Res. Technol..

[193] Villa C., Costa J., Oliveira M.B.P., Mafra I. (2018). Bovine Milk Allergens: A Comprehensive Review. Compr. Rev. Food Sci. Food Saf..

[194] Matsumoto N., Okochi M., Matsushima M., Kato R., Takase T., Yoshida Y., Kawase M., Isobe K.-I., Kawabe T., Honda H. (2009). Peptide array-based analysis of the specific IgE and IgG4 in cow’s milk allergens and its use in allergy evaluation. Peptides.

[195] Fiocchi A., Brozek J., Schünemann H., Bahna S.L., von Berg A., Beyer K., Bozzola M., Bradsher J., Compalati E., Ebisawa M. (2010). World allergy organization (WAO) diagnosis and rationale for action against cow’s milk allergy (DRACMA) guidelines. World Allergy Organ. J..

[196] Martorell-Aragonés A., Echeverría-Zudaire L., Alonso-Lebrero E., Boné-Calvo J., Martín-Muñoz M., Nevot-Falcó S., Piquer-Gibert M., Valdesoiro-Navarrete L. (2015). Position document: IgE-mediated cow’s milk allergy. Allergol. Immunopathol..

[197] Buttar H.S., Bagwe S.M., Bhullar S.K., Kaur G. (2017). Health Benefits of Bovine Colostrum in Children and Adults. Dairy in Human Health and Disease Across the Lifespan.

